# The Effect of Raltegravir Intensification on Low-level Residual Viremia in HIV-Infected Patients on Antiretroviral Therapy: A Randomized Controlled Trial

**DOI:** 10.1371/journal.pmed.1000321

**Published:** 2010-08-10

**Authors:** Rajesh T. Gandhi, Lu Zheng, Ronald J. Bosch, Ellen S. Chan, David M. Margolis, Sarah Read, Beatrice Kallungal, Sarah Palmer, Kathy Medvik, Michael M. Lederman, Nadia Alatrakchi, Jeffrey M. Jacobson, Ann Wiegand, Mary Kearney, John M. Coffin, John W. Mellors, Joseph J. Eron, on behalf of the AIDS Clinical Trials Group A5244 team

**Affiliations:** 1 Massachusetts General Hospital and Ragon Institute, Boston, Massachusetts, United States of America; 2 Harvard School of Public Health, Boston, Massachusetts, United States of America; 3 University of North Carolina, Chapel Hill, North Carolina, United States of America; 4 National Institute of Allergy and Infectious Diseases, Bethesda, Maryland, United States of America; 5 Social & Scientific Systems, Silver Spring, Maryland, United States of America; 6 Karolinska Institute, Stockholm, Sweden; 7 Case Western Reserve University, Cleveland, Ohio, United States of America; 8 Beth Israel Deaconess Medical Center, Boston, Massachusetts, United States of America; 9 Drexel University, Philadelphia, Pennsylvania, United States of America; 10 National Cancer Institute, Frederick, Maryland, United States of America; 11 Tufts University, Boston, Massachusetts, United States of America; 12 University of Pittsburgh, Pittsburgh, Pennsylvania, United States of America

## Abstract

**Background:**

Most HIV-1-infected patients on effective antiretroviral therapy (ART) with plasma HIV-1 RNA levels below the detection limits of commercial assays have residual viremia measurable by more sensitive methods. We assessed whether adding raltegravir lowered the level of residual viremia in such patients.

**Methods and Findings:**

Patients receiving ART who had plasma HIV-1 RNA levels below 50 copies/mL but detectable viremia by single copy assay (SCA) were randomized to add either raltegravir or placebo to their ART regimen for 12 weeks; patients then crossed-over to the other therapy for an additional 12 weeks while continuing pre-study ART. The primary endpoint was the plasma HIV-1 RNA by SCA averaged between weeks 10 and 12 (10/12) compared between treatment groups. Fifty-three patients were enrolled. The median screening HIV-1 RNA was 1.7 copies/mL. The HIV-1 RNA level at weeks 10/12 did not differ significantly between the raltegravir-intensified (*n* = 25) and the placebo (*n* = 24) groups (median 1.2 versus 1.7 copies/mL, *p* = 0.55, Wilcoxon rank sum test), nor did the change in HIV-1 RNA level from baseline to week 10/12 (median −0.2 and −0.1 copies/mL, *p* = 0.71, Wilcoxon rank sum test). There was also no significant change in HIV-1 RNA level from weeks 10/12 to weeks 22/24 after patients crossed-over. There was a greater CD4 cell count increase from baseline to week 12 in the raltegravir-intensified group compared with the placebo group (+42 versus −44 cells/mm^3^, *p* = 0.082, Wilcoxon rank sum test), which reversed after the cross-over. This CD4 cell count change was not associated with an effect of raltegravir intensification on markers of CD4 or CD8 cell activation in blood.

**Conclusion:**

In this randomized, double-blind cross-over study, 12 weeks of raltegravir intensification did not demonstrably reduce low-level plasma viremia in patients on currently recommended ART. This finding suggests that residual viremia does not arise from ongoing cycles of HIV-1 replication and infection of new cells. New therapeutic strategies to eliminate reservoirs that produce residual viremia will be required to eradicate HIV-1 infection.

**Trial Registration:**

ClinicalTrials.gov NCT00515827

*Please see later in the article for the Editors' Summary*

## Abstract 2

In a double-blind trial, Rajesh Gandhi and colleagues detect no significant reduction in viral load after people with low-level HIV viremia added an integrase inhibitor to their treatment regimen.

## Editors' Summary

**Background:**

Acquired immunodeficiency syndrome (AIDS) has killed about 25 million people since 1981 and more than 30 million people are now infected with the human immunodeficiency virus (HIV), which causes AIDS. HIV is a retrovirus—its genetic blueprint is made of ribonucleic acid (RNA). HIV infects human immune system cells and destroys them, leaving infected individuals susceptible to other infections. Early during the AIDS epidemic, most HIV-positive people died within ten years of infection. Then, in 1996, effective antiretroviral therapy (ART) was developed. ART consists of combinations of drugs that prevent viral replication by inhibiting essential viral enzymes such as reverse transcriptase (the enzyme that makes a DNA copy of the viral RNA; a viral enzyme called integrase inserts this DNA copy into the host cell DNA where it remains dormant until the host cell is activated) and protease (an enzyme needed for the production of new viral particles, which are released into the blood stream). Now, in industrialized countries, the life expectancy of HIV-infected patients treated with ART is similar to that of people with diabetes and other chronic conditions.

**Why Was This Study Done?:**

Although ART can reduce the number of viral RNA copies in the plasma (the liquid portion of blood) of HIV-positive patients to less than 50 copies/mL (the limit of detection of commercial assays), it is does not eradicate HIV. When very sensitive assays are used to detect viral RNA (for example, the “single copy assay” or SCA), most patients on ART have one copy or more of HIV RNA per mL of plasma. The origin of this low-level residual viremia (virus in the blood) is controversial. Residual viremia could arise from ongoing cycles of viral replication, in which case intensification of ART should reduce it. Alternatively, residual viremia could be due to HIV release from stable reservoirs such as latently infected resting immune system cells, in which case intensification of ART should have no effect on residual viremia. In this randomized, controlled trial (a study in which randomly selected groups of patients are given different treatments and the effects of these treatments compared), the researchers assess whether the addition of raltegravir (a drug that inhibits HIV integrase) to standard ART has any effect on residual viremia.

**What Did the Researchers Do and Find?:**

The researchers enrolled 53 HIV-positive patients who had been receiving ART containing several reverse transcriptase inhibitors and, in some cases, a protease inhibitor for at least 12 months and who had a plasma HIV RNA level below 50 copies/mL but detectable viremia by SCA. The patients were randomly assigned to receive either raltegravir or a dummy drug (placebo) in addition to their normal ART for 12 weeks. They were then crossed-over (swapped) to the other therapy for a further 12 weeks. At baseline, the trial participants had an average plasma HIV RNA level of 1.7 copies/mL. The HIV RNA level at weeks 10/12 (the average of SCA results at 10 and 12 weeks) was similar in the raltegravir group and in the placebo group and did not differ significantly from this baseline level. There was also no significant change in plasma HIV RNA levels from weeks 10/12 to weeks 22/24 after the patients crossed-over between treatment groups.

**What Do These Findings Mean?:**

In this randomized, cross-over study, raltegravir intensification of ART for 12 weeks did not demonstrably reduce low-level residual viremia in HIV-positive patients receiving standard ART. It is possible that 12 weeks is too short a time to see an effect of raltegravir on residual viremia. Furthermore, although this is one of the biggest trials of this type done to date, it might be that insufficient patients were included in the trial to detect a subtle effect of raltegravir on residual viremia. Nevertheless, these findings argue against the hypothesis that residual viremia arises from ongoing cycles of viral replication and the infection of new cells. Instead, they suggest that residual viremia might be due to the release of HIV from stable reservoirs. If so, new therapeutic strategies designed to eliminate these reservoirs of latently infected cells will be required to cure HIV infection.

**Additional Information:**

Please access these Web sites via the online version of this summary at http://dx.doi.org/10.1371/journal.pmed.1000321.

- Information is available from the US National Institute of Allergy and infectious diseases on HIV infection and AIDS, and on the treatment of HIV
- HIV InSite has comprehensive information on all aspects of HIV/AIDS, including information on antiretroviral therapies
- Information is available from Avert, an international AIDS charity on many aspects of HIV/AIDS, including the treatment of HIV and AIDS (in English and Spanish)
- MedlinePlus has links to further resources on AIDS and on AIDS medicines (in English and Spanish)

## Introduction

Although currently recommended antiretroviral therapy (ART) lowers plasma HIV-1 RNA levels to below the detection limit of commercial assays (generally <50 copies/mL), most patients have persistent low-level viremia when tested with more sensitive methods [1]. In fact, using a real-time PCR assay that detects a single copy of HIV-1 RNA, more than 80% of patients on combination ART had viremia of one copy/mL or more [2]. This residual viremia does not measurably decay after up to 7 years of ART [3].

The causes and clinical implications of persistent low-level viremia in patients on ART are controversial (reviewed in [4]). Residual plasma viremia may arise from ongoing cycles of viral replication and infection of new cells, virus release from stable reservoirs, or both. If persistent viremia in patients who are receiving ART is mainly due to viral replication, intensifying therapy by adding a new, potent antiretroviral drug that blocks new cycles of replication should reduce residual viremia and prevent repletion of viral reservoirs, a crucial step toward HIV-1 eradication. Alternatively, if residual viremia is primarily due to HIV-1 release from stable reservoirs, such as latently infected resting CD4 memory cells or other long-lived cells, then treatment intensification with drugs that block only new cycles of viral replication would not be expected to reduce persistent viremia. Indeed, prior intensification studies of patients on suppressive three-drug antiretroviral regimens did not observe reductions in plasma HIV-1 RNA levels after the addition of antiretroviral drugs that block new replication cycles, including inhibitors of reverse transcriptase (RT), protease, and integrase [5],[6]. However, these studies were small, single-arm trials of short duration that were not randomized or placebo-controlled and had limited power to detect changes in residual viremia. Larger, randomized, and placebo-controlled trials are needed to more definitely assess the impact of treatment intensification on residual viremia.

Raltegravir, the first U.S. Food and Drug Administration-approved HIV-1 integrase inhibitor, is an attractive agent to study the effects of treatment intensification on residual viremia. The drug is a potent inhibitor of HIV-1 replication [7],[8] and blocks the virus through a mechanism different from those of other commonly prescribed antiretroviral medications, such as RT and protease inhibitors. If low-level residual viremia is the result of incomplete inhibition of HIV-1 replication by RT and protease inhibitors, then blocking a third viral enzyme with raltegravir might reduce HIV-1 RNA levels.

To assess whether raltegravir intensification lowers residual viremia in patients on combination ART, we conducted a multi-center randomized, double-blind placebo-controlled cross-over study (AIDS Clinical Trials Group study A5244).

## Methods

This study was conducted according to the principles expressed in the Declaration of Helsinki. The study (Text S1 and S2) was approved by the Institutional Review Boards of all institutions at which patients were enrolled. All patients provided written informed consent for study participation, the collection of samples and subsequent analysis. The NCT number for this study is NCT00515827.

The main inclusion criteria for the study (Text S1) were: (1) HIV-1 infected adults receiving ART for at least 12 months with two or more nucleoside reverse transcriptase inhibitors (NRTI) and either a ritonavir-boosted protease inhibitor (PI) or a non-nucleoside reverse transcriptase inhibitor (NNRTI); (2) plasma HIV-1 RNA levels below limits of detection for ≥6 months using commercial assays; and (3) CD4 cell count ≥200/mm^3^. Participants were also required to have documentation of a pre-ART HIV-1 RNA level of >100,000 copies/mL because detectable residual viremia is more likely in individuals with higher pre-treatment HIV-1 RNA levels [2]. Patients were excluded if they had a history of documented virologic failure while on ART. All participants were integrase inhibitor-naïve.

**Figure 1:**
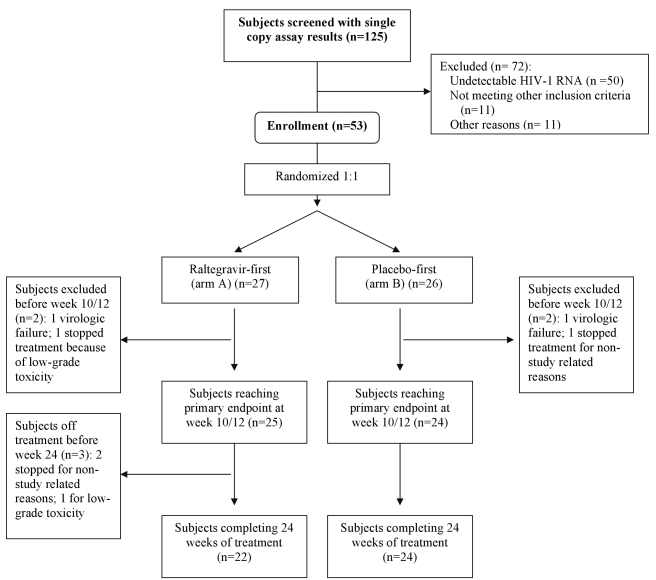
Participant disposition.

Patients who met the above criteria underwent testing with a real-time PCR assay that can detect a single copy of HIV-1 RNA in a plasma sample (Figure 1). The quantification limit is determined by the volume of plasma tested. Approximately 50% of the plasma volume is used for HIV-1 RNA quantification and the remainder for assay controls; e.g. if 10 mL of plasma is tested, the lower limit of quantification (LLQ) is 0.2 copies/mL [9]. At the time of screening, the plasma volume assayed was ≥7 mL in 97% of patients and ≥10 mL in 75% of patients. Participants who had detectable screening HIV-1 RNA measured by this single copy assay (SCA) were eligible for the study.

**Figure 2:**
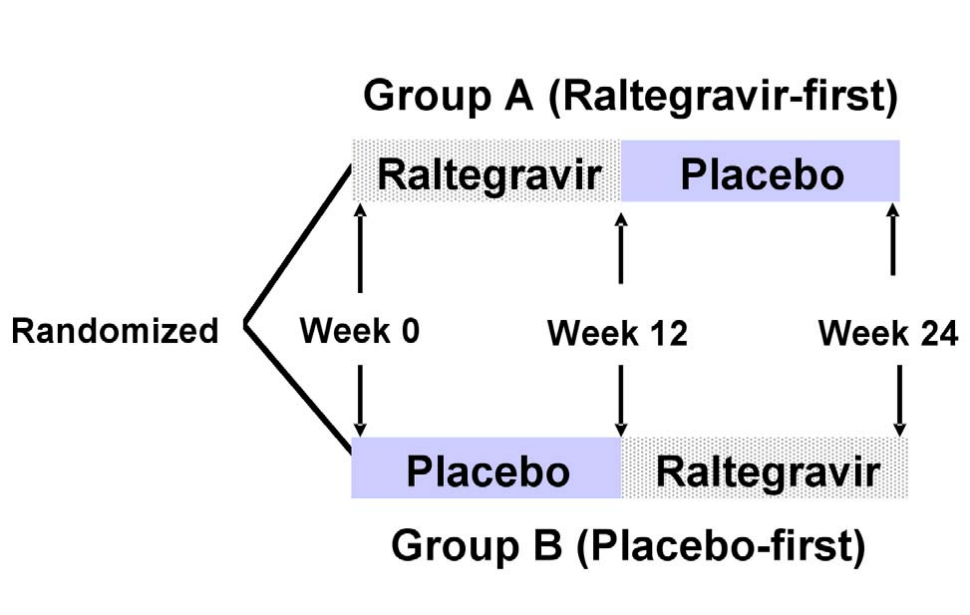
Study schema.

Eligible patients were randomized with equal probability to either immediate-intensification (group A) or delayed-intensification (group B) with raltegravir, using permuted blocks, stratified by whether they were on a PI- or NNRTI-containing entry regimen (Figure 2). Participants in the immediate-intensification group (raltegravir-first) added raltegravir 400 mg twice daily to their entry regimen at week 0. At week 12, participants in this group stopped raltegravir and added matching placebo twice daily for 12 more weeks. Patients in the delayed intensification group (placebo-first) added placebo twice daily to their entry regimen at week 0. At week 12, those in this group stopped placebo and added raltegravir 400 mg twice daily for 12 more weeks. The planned duration of this double-blind study was 24 weeks. To estimate participant adherence to study drug, pill counts were performed at weeks 2, 4, 10, 12, 14, 16, 22, and 24. Adverse events were graded using the DAIDS Toxicity Grading Tables (available at http://rsc.tech-res.com/safetyandpharmacovigilance/) on a scale of 1 to 4 that signifies severity ranging from mild to life-threatening.

SCA testing was performed at pre-entry, entry, and weeks 10, 12, 22, and 24. Samples from these time points were assayed in a batch for each patient (i.e., all samples from an individual participant were assayed in a single test run). The plasma volume assayed was ≥7 mL in ≥90% of samples (range at the different time points, 90%–94%) and ≥10 mL in >83% of samples (range at the different time points, 84%–94%). The HIV-1 RNA log_10_ copies/mL values by SCA were averaged at pre-entry and entry (baseline [average of the pre-entry and entry measurements]), weeks 10 and 12 (10/12), and weeks 22 and 24 (22/24). CD4 and CD8 cell counts were assessed by flow cytometry at pre-entry and entry (the baseline value was the average of the two measurements) and again at weeks 12 and 24. CD4 and CD8 cell activation in the blood was assessed on cryopreserved cells at pre-entry and entry (the baseline value was the average of the two measurements) and at weeks 12 and 24. The percentage of CD4 and CD8 cells that expressed the activation markers CD38 and human leukocyte antigen (HLA)-DR was determined using phycoerythrin-conjugated anti-CD38, fluorescein isothiocyanate–conjugated anti-HLA–DR, allophycocyanin-conjugated anti-CD4, and peridinin chlorophyll protein-conjugated anti-CD8 monoclonal antibodies and analyzed by flow cytometry.

The primary objective of the study was to compare the HIV-1 RNA level by SCA at weeks 10/12 in patients who added raltegravir to a PI- or NNRTI-containing regimen to the level in patients who added placebo to a PI- or NNRTI-containing regimen. We conducted an on-treatment analysis and excluded patients who had confirmed virologic rebound to >50 copies/mL of HIV-1 RNA. The target enrollment for the study was 50 participants (25 in each group). From prior data on assay and biologic variation in residual HIV-1 RNA levels [2], we estimated that 18 evaluable participants in each treatment group would provide 80% power to detect a 0.5 log_10_ difference (1 standard deviation effect size) in the HIV-1 RNA level at weeks 10/12 between the two treatment groups using a Wilcoxon rank-sum two-sided test at a 0.05 level. We increased the target enrollment to 25 in each group with the expectation that some participants would discontinue study drug prior to week 12 and that treatment intensification might increase the proportion of participants who have HIV-1 RNA levels below the LLQ by SCA at weeks 10/12, which would affect statistical power. When averaging the week 10 and 12 measurements, if one or both measurements were below the SCA lower assay limits, the LLQ was used to compute the average and the primary endpoint was treated as below the averaged value. The primary endpoint was analyzed using the censored-data version of the Wilcoxon rank-sum test, which accommodates the fact that participants had different SCA lower limits due to varying plasma volumes. For analyses of secondary endpoints (such as the change in HIV-1 RNA value by SCA, CD4 and CD8 cell counts, and activation markers), treatment arms were compared using exact Wilcoxon rank sum tests for continuous endpoints or Fisher's exact tests for proportions. The within-participant effect of intensification was tested by Wilcoxon signed rank test. Confidence intervals (CIs) were estimated using Hodges and Lehmann method. Associations between responses and predictors were assessed using rank-based Spearman correlations. Reported *p*-values were two-sided without adjustment for multiple comparisons.

## Results

### Baseline Characteristics

One hundred twenty-five patients were screened for the study, of whom 75 (60%) had detectable plasma HIV-1 RNA by SCA (Figure 1). Fifty-three patients from 19 different clinical research sites enrolled in the randomized clinical trial. Of the 22 patients with detectable viremia who did not enroll, 11 were found to be ineligible (most commonly because a pre-ART HIV-1 RNA value was not available or the patient did not otherwise meet the eligibility criteria) and 11 did not enroll for other reasons (in most cases the reason was not specified). There were no significant differences in baseline characteristics (age, race, sex, screening CD4 cell count, entry regimen, or time since first undetectable HIV-1 RNA value) between the 22 patients with a detectable HIV-1 RNA by SCA who did not enroll and the 53 patients who enrolled.

The baseline characteristics of the 53 patients who enrolled in the study are summarized in Table 1. The median age was 49 years and 91% were male. Sixty-six percent of patients were on an NNRTI-containing regimen and 34% were on a PI-containing regimen. The baseline CD4 cell count was 589/mm^3^, and the median time since the first undetectable HIV-1 RNA level on a commercial assay was 5.5 years. The median (quartile [Q] 1, Q3) screening HIV-1 RNA level by SCA was 1.7 copies/mL (0.6, 2.9). Participants in the raltegravir-first and placebo-first groups were well-balanced with respect to these baseline characteristics. The median screening HIV-1 RNA by SCA was 1.2 copies/mL in the raltegravir-first group and 1.9 copies/mL in the placebo-first group; the interquartile ranges for the median HIV-1 RNA values in the two groups overlap (Table 1). In addition, even though all participants had a detectable HIV-1 RNA by SCA at screening, 22% had a value below the lower limits of quantification at either study entry or pre-entry; there was no statistically significant difference in the frequency of this finding between the two study groups.

**Table 1:** Baseline characteristics.

Characteristic	Overall (*n* = 53)	Raltegravir-First (Group A) (*n* = 27)	Placebo-First (Group B) (*n* = 26)
**Age, median (years)**	49	51	47
**Male sex (%)**	48 (91%)	24 (89%)	24 (92%)
**Race/ethnicity**			
White non-Hispanic	36 (68%)	20 (74%)	16 (62%)
Black non-Hispanic	9 (17%)	5 (19%)	4 (15%)
Hispanic	7 (13%)	2 (7%)	5 (19%)
Not-reported	1 (2%)	0 (0%)	1 (4%)
**Background entry regimen**			
PI-containing	18 (34%)	9 (33%)	9 (35%)
NNRTI-containing	35 (66%)	18 (67%)	17 (65%)
**Baseline CD4 count (cells/mm^3^), median** (Q1, Q3)	589 (452, 751)	538 (401, 717)	613 (454, 833)
**Years since first undetectable HIV-1 RNA value**, median (Q1, Q3)	5.5 (3.5, 7.0)	4.8 (3.2, 7.9)	5.7 (3.7, 7.0)
**Screening HIV-1 RNA by SCA** (copies/mL), median (Q1, Q3)	1.7 (0.6, 2.9)	1.2 (0.2, 3.1)	1.9 (0.8, 2.9)

Quartile (Q)1 and Q3 are 25^th^ and 75^th^ percentiles. NNRTI, non-nucleoside reverse transcriptase inhibitor; PI, protease inhibitor; SCA, single-copy assay.

### Safety and Tolerability of Raltegravir Intensification

Of the 53 patients who initiated study treatment, 49 (92%) contributed data to the primary endpoint at weeks 10/12 and 46 (87%) completed all 24 weeks of the study treatment (Figure 1). In those who had pill counts to estimate adherence, we found that the average adherence across participants ranged from 94% to 97% at different study time points. One participant in each group stopped treatment because of confirmed virologic failure (2 consecutive HIV-1 RNA levels >50 copies/mL); both had HIV-1 RNA levels of <250 copies/mL and subsequently achieved a HIV-1 RNA <50 copies/mL without changing their entry regimen (one participant had genotypic resistance testing performed on samples drawn during the times he had detectable viremia, but the virus did not have integrase gene mutations or any other new resistance mutations). The rate of premature study discontinuation was not appreciably different between the two treatment groups (two participants in the raltegravir-first group, one in the placebo-first group). There was no statistically significant difference between the groups in the rate of grade 2 or higher signs and symptoms or grade 3 or higher lab abnormalities. Overall, the study drug was well-tolerated.

### Effect of Raltegravir Intensification on Plasma HIV-1 RNA Level

**Figure 3:**
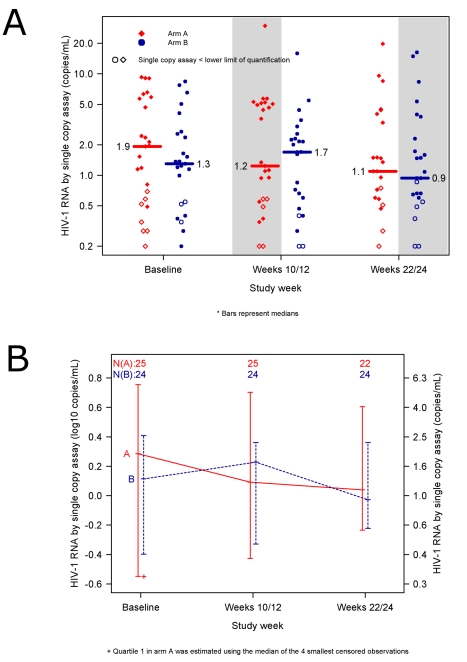
No reduction in low-level residual viremia after raltegravir intensification. (A) HIV-1 RNA values in patients in the raltegravir-first group (red diamonds) compared with participants in the placebo-first group (blue circles). Shaded areas designate time periods during which participants were receiving raltegravir intensification. Open symbols represent results for which one or both single copy assay (SCA) determinations were below the lower limit of quantification. Median SCA values at each time point are reported (horizontal bars). The average HIV-1 RNA level at week 10/12—the primary endpoint of the study—did not differ significantly between the raltegravir-first and the placebo-first groups: median 1.2 vs. 1.7 copies/mL (*p* = 0.55, Wilcoxon rank sum test). (B) Median HIV-1 RNA levels in participants adding raltegravir-first at week 0 and then crossing over to placebo at week 12 (red solid line) compared with participants adding placebo-first at week 0 and then crossing over to raltegravir at week 12 (blue dashed line). The lines connect medians. The bars represent interquartile ranges (25^th^ and 75^th^ percentiles).

The primary objective of the study was to compare the HIV-1 RNA level by SCA at weeks 10/12 in participants who added raltegravir to a PI- or NNRTI-containing regimen to the level in those who added placebo to a PI- or NNRTI-containing regimen. The average HIV-1 RNA level at week 10/12 did not differ significantly between the raltegravir-intensified (*n* = 25) and the placebo group (*n* = 24): median 1.2 (0.1 log_10_) versus 1.7 (0.2 log_10_) copies/mL (*p* = 0.55, Wilcoxon rank sum test, 95% CI for the difference between the groups: −0.4 log_10_ to 0.3 log_10_ copies/mL) (Figure 3A). A similar result was obtained in an intent-to-treat analysis, which included all participants with week 10/12 data regardless of treatment status or virologic failure (*n* = 51). In the intent-to-treat analysis, the median HIV-1 RNA at week 10/12 in the raltegravir-first group was 1.2 (0.1 log_10_) copies/mL and in the placebo group was 1.7 (0.2 log_10_) copies/mL; there was no statistically significant difference between the two groups (*p* = 0.73, Wilcoxon rank sum test, 95% CI for the difference between the groups: −0.4 to 0.4 log_10_ copies/mL). The absence of a demonstrable difference in HIV-1 RNA level at week 10/12 between the two treatment groups was also seen in planned sensitivity analyses that adjusted for ART regimen at entry, baseline HIV-1 RNA by SCA and/or baseline CD4 cell count (using regression models for censored data) (*p*-values 0.71 to 0.81, Wald test, data not shown).

We also assessed the difference between the pre-intensification and post-intensification SCA values in the two treatment groups. The change in HIV-1 RNA from baseline to weeks 10/12 did not differ significantly between the two treatment groups (median −0.2 and −0.1 copies/mL, *p* = 0.71, Wilcoxon rank sum test) (for this comparison, measurements below the LLQ were imputed a value of the LLQ divided by 2) (Figure 3B). The proportion of patients who had one or more HIV-1 RNA levels below the LLQ at week 10/12 did not differ between the two groups (20% versus 13%, *p* = 0.70, Fisher's exact test). In a protocol-specified secondary analysis, we also evaluated the change in HIV-1 RNA level from the pre-intensification level (for the raltegravir-first group: average of pre-entry and entry values; for the placebo-first group: average of week 10 and 12 values) to the post-intensification level (raltegravir-first group: weeks 10/12; placebo-first group: weeks 22/24). The median change in HIV-1 RNA level from pre-intensification to post-intensification was 0.0 (−0.07 log_10_) copies/mL (95% CI of −0.5 to 0.3 copies/mL or −0.1 to 0.1 log_10_ copies/mL). That is, we can exclude, with 95% confidence, a median decline from baseline HIV-1 RNA during intensification of more than 24% (>0.1 log_10_ copies/mL). In our study population, we can exclude a median reduction in HIV-1 RNA during intensification of more than 0.5 copies/mL. The proportion of participants who had either an increase or no change versus a decrease in HIV-1 RNA levels during the intensification period did not differ significantly between treatment groups (unpublished data).

In addition, the cross-over design allowed us to evaluate whether there was an increase in HIV-1 RNA level after discontinuation of raltegravir by patients in the raltegravir-first group, or a decrease in HIV-1 RNA level after addition of raltegravir by those in the group that received placebo first. There was no significant change in HIV-1 RNA level after participants crossed-over from raltegravir to placebo or from placebo to raltegravir (unpublished data).

### Effect of Raltegravir Intensification on CD4 and CD8 Cell Counts

We also examined the effect of raltegravir intensification on CD4 and CD8 cell counts, which were measured at baseline and at weeks 12 and 24 (Table 2). There was a greater CD4 count increase from baseline to week 12 in the raltegravir-first group compared with the placebo-first group (median +42 cells/mm^3^ vs. −44 cells/mm^3^), but this difference was not statistically significant (*p* = 0.082, Wilcoxon rank sum test). After the cross-over, the change in CD4 cell count reversed in the two groups: the CD4 count in the group that switched to placebo dropped by a median of 26 cells/mm^3^, whereas it increased by 54 cells/mm^3^ in the group that added raltegravir (*p* = 0.066 for the comparison, Wilcoxon rank sum test). When the treatment groups were pooled, the median CD4 count change was an increase of 42 cells/mm^3^ during raltegravir intensification (95% CI −2 to 57 cells/mm^3^). There was also no significant change in CD8 cell count during raltegravir intensification.

**Table 2:** CD4 cell count changes in participants receiving immediate or delayed intensification with raltegravir.

Measure	Immediate Intensification (Raltegravir-First)	Delayed Intensification (Placebo-First)	*p*-Value*
Baseline CD4 count (cells/mm^3^) median (Q1, Q3)	538 (401, 717)	613 (454, 833)	—
Change from baseline to week 12 (cells/mm^3^) median (Q1, Q3)	+42 (−52, 80)	−44 (−117, 49)	0.082
Change from week 12 to week 24 (cells/mm^3^) median (Q1, Q3)	−26 (−54, 44)	+54 (−52, 144)	0.066

Quartile (Q)1 and Q3 are 25^th^ and 75^th^ percentiles.

*Wilcoxon rank sum test.

### Effect of Raltegravir Intensification on T Cell Activation

Because HIV-1 replication is associated with elevated T cell activation, we looked for a relationship between baseline activation and HIV-1 RNA level. CD4 and CD8 cell activation was determined by measuring the percentage of cells that expressed both CD38 and HLA-DR. There was no association between baseline HIV-1 RNA value by SCA and baseline T cell activation (for example, *r* = 0.08 for the correlation between HIV-1 RNA and CD4+/CD38+/HLA-DR+ percentage; *r* = 0.01 for the correlation between HIV-1 RNA and CD8+/CD38+/HLA-DR+ percentage; both *p*-values>0.6, Spearman rank correlation).

In addition, we evaluated the association between baseline T cell activation and other participant characteristics. There was no correlation between age and either CD4+/CD38+/HLA-DR+ or CD8+/CD38+/HLA-DR+ percentage. The duration of virologic suppression and pre-ART HIV-1 RNA level were also not correlated with T cell activation.

We then examined whether raltegravir intensification affected the level of CD8 and CD4 cell activation (Table 3), as might be expected if adding the drug inhibits ongoing low-level viral replication that is inducing T cell activation. At baseline, patients in the raltegravir-first group had a median of 11% CD8+/CD38+/HLA-DR+ cells and those in the placebo-first group had a median of 16% CD8+/CD38+/HLA-DR+ cells. We found no statistically significant difference in the CD8+/CD38+/HLA-DR+ percentage between the two treatment groups at week 12 or 24. There was also no significant difference between the two treatment groups in the change in CD8+/CD38+/HLA-DR+ percentage from baseline to week 12 (−1% in the raltegravir-first group versus 0% in the placebo-first group, *p* = 0.50, Wilcoxon rank sum test) or from week 12 to week 24 (+2% in the group that crossed-over to add placebo versus −3% in the group that crossed-over to add raltegravir, *p* = 0.35, Wilcoxon rank sum test). We performed a similar analysis of the effect of raltegravir intensification on CD4 cell activation. At baseline, participants in both the raltegravir- and placebo-first groups had a median of 7% CD4+/CD38+/HLA-DR+ cells. We found no statistically significant difference in CD4+/CD38+/HLA-DR+ percentage between the treatment groups at week 12 or 24. There was also no significant difference between the two treatment groups in the change in CD4+/CD38+/HLA-DR+ percentage from baseline to week 12 or from week 12 to week 24.

**Table 3:** Change in CD4 and CD8 activation by treatment group.

Measure	Immediate Intensification (Raltegravir-First)	Delayed Intensification (Placebo-First)	*p*-Value*
**CD8+CD38+HLA-DR+ Percentage**			
Baseline, median (Q1, Q3)	11 (8, 20)	16 (11, 20)	—
Median change (Q1, Q3), baseline to week 12	*−1 (−2, 2)*	0 (−2, 5)	0.50
Median change (Q1, Q3), week 12 to week 24	2 (−1, 5)	*−3 (−6, 5)*	0.35
**CD4+CD38+HLA-DR+ Percentage**			
Baseline, median (Q1, Q3)	7 (5, 9)	7 (6, 7)	—
Median change (Q1, Q3), baseline to week 12	*−1 (−2, 1)*	0 (−1, 2)	0.20
Median change (Q1, Q3), week 12 to week 24	1 (−1, 3)	*0 (−3, 2)*	0.18
**CD8+CD38+ Percentage**			
Baseline, median (Q1, Q3)	39 (27, 50)	39 (34, 44)	—
Median change (Q1, Q3), baseline to week 12	*−3 (−11, 0)*	1 (−3, 7)	0.053
Median change (Q1, Q3), week 12 to week 24	0 (−14, 11)	*0 (−14, 3)*	0.36
**CD4+CD38+Percentage**			
Baseline, median (Q1, Q3)	58 (49, 74)	60 (50, 67)	—
Median change (Q1, Q3), baseline to week 12	*−5 (−11, 0)*	−1 (−6, 4)	0.10
Median change (Q1, Q3), week 12 to week 24	1 (−17, 11)	*−2 (−14, 4)*	0.82

Our primary measure of CD8 activation was the CD8+CD38+HLA-DR+ percentage and of CD4 activation was the CD4+CD38+HLA-DR+ percentage (see text). Italicized values designate a measurement was made during the period of raltegravir intensification.

Quartile (Q)1 and Q3 are 25^th^ and 75^th^ percentiles.

*Wilcoxon rank sum test.

We also evaluated whether raltegravir intensification reduced the percentage of CD4 and CD8 cells that expressed CD38, as might be expected if viral replication was decreased, but CD38 expression was not our primary measure of immune activation because naïve T cells can express this marker in the absence of activation [10],[11],[12]. At baseline, there was an inverse correlation between age and CD4+/CD38+ percentage (*r* = −0.31, *p* = 0.03, Spearman rank correlation). There was no association between age and CD8+/CD38+ percentage (*r* = −0.24, *p* = 0.10, Spearman rank correlation). The median change in CD8+/CD38+ from pre-intensification to post-intensification—when data from the two treatment groups were pooled—was −2.0% (95% CI −10% to 0%) (*p* = 0.046, Wilcoxon signed rank test). Although there was a marginally significant difference between the groups in the change in CD8+/CD38+ percent from baseline to week 12—there was a median of a 3% decline in the group that added raltegravir versus a 1% increase in the group that added placebo (*p* = 0.053 for the comparison, Wilcoxon rank sum test)—the change in CD8+/CD38+ percentage from week 12 to 24 (after the cross-over) did not differ between the treatment groups (Table 3). We performed a similar analysis of the effect of intensification on the percentage of CD4 cells that express CD38. When the two treatment groups were pooled, the median percentage of cells expressing CD38 decreased by 4% during the period of raltegravir intensification (95% CI −9.5 to −0.5%, *p* = 0.030, Wilcoxon signed rank test). However, there was no significant difference between the groups in the change in CD4+/CD38+ percentage from baseline to week 12 (median −5% in the raltegravir-first group versus −1% in the placebo-first group, *p* = 0.10, Wilcoxon rank sum test) or from week 12 to 24 (+1% in the group that crossed-over to add placebo versus −2% in the group that crossed-over to add raltegravir, *p* = 0.82, Wilcoxon rank sum test) (Table 3).

Finally, to determine whether the trend toward an increase in CD4 cell count during raltegravir intensification described above could be explained by an effect on T cell activation, we analyzed the relationship between change in activation markers and increase in CD4 cell count. We found no associations between the changes in CD4 or CD8 CD38+/HLA-DR+ percentage or CD38+ percentage and the trend towards a CD4 cell count increase during raltegravir intensification (unpublished data).

## Discussion

In this randomized, double-blind, placebo-controlled cross-over study, 12 weeks of raltegravir intensification did not demonstrably reduce residual viremia in patients on PI- or NNRTI-containing ART. Although there was a trend toward an increased CD4 cell count during the period of raltegravir intensification, this change was not associated with an effect of raltegravir on T cell activation. CD4 and CD8 cell activation levels in blood, as determined by the percentage of CD38+/HLA-DR+ cells, were not associated with the baseline level of residual viremia, and we did not find evidence that raltegravir intensification reduced T cell activation by this measure.

The findings of this study are consistent with the results of two smaller, single-arm trials. In a study of treatment intensification with 4–8 weeks of efavirenz, atazanavir/ritonavir or lopinavir/ritonavir in nine HIV-1 infected patients, there was no effect of adding the additional agent on HIV-1 RNA level measured by SCA [5]. Similarly, in a study of ten patients, there was no reduction in HIV-1 RNA level during 4 weeks of raltegravir intensification [6]. In contrast, an earlier trial found that adding abacavir reduced the level of HIV-1 RNA in patients receiving indinavir plus efavirenz: of five patients with detectable viremia on this two-drug regimen, four had a decline in viral RNA level after adding abacavir [13]. However, the two-drug regimen of indinavir plus efavirenz in this trial may not have blocked viral replication as effectively as current three-drug combination ART, which could explain why adding an agent lowered HIV-1 RNA in the early study but not in more recent treatment intensification trials. The results of the current trial are also consistent with other studies which find that there is no evidence for substantive HIV-1 replication in patients on three-drug combination ART, as assessed by the absence of viral evolution and the lack of accumulation of drug resistance mutations [14],[15].

A recently published study of raltegravir intensification has been interpreted as showing that ongoing viral replication occurs in a subset of patients on combination ART. Buzon et al. found that 13 of 45 (29%) patients on suppressive ART who were randomized to add raltegravir had a transient increase in 2-LTR circles—an episomal form of HIV DNA—which the authors interpret as reflecting persistent replication [16]. Although the findings of this study are important and provocative, its implications for understanding the effect of intensification on viral replication are uncertain for several reasons. First, of the 13 participants in whom 2-LTR circles were affected by raltegravir, five had detectable circles even before adding the drug, whereas none of the control patients had detectable 2-LTR circles at baseline, suggesting that the treatment groups may have differed at the outset of the study. Second, patients with prior antiretroviral treatment failure, who might have less complete viral suppression, were enrolled in the Buzon study, whereas such individuals were excluded from our study. Third, it is not clear why only a subset of individuals had an increase in 2-LTR circles. Patients who had an increase in 2-LTR circles may not have been on ART long enough to have achieved stable virologic suppression: individuals who became 2-LTR^+^ had received suppressive ART for a shorter time (mean 3.5 ± standard deviation (SD) of 2.5 years) than those who did not become 2-LTR^+^ (mean 5.2 ± SD 2.9 years) (*p* = 0.075). In our study, patients had been on suppressive ART for a mean of 5.6 ± SD 3 years (median of 5.5 years). The fact that increases in 2-LTR circles occurred mainly in patients who were on protease inhibitor-containing regimens suggests a mechanistic explanation independent of ongoing full cycles of viral replication, e.g., that the accumulation of 2-LTR circles is related to the stage at which different antiretroviral agents block HIV-1 rather than a reflection of complete cycles of viral replication. Finally, in the Buzon study, there was no effect of raltegravir intensification on plasma HIV-1 RNA levels, as measured by a single copy assay; this finding is the same as the result we obtained. The absence of an effect on plasma HIV-1 RNA implies that raltegravir intensification does not reduce ongoing viral replication, unless the virus is replicating in a compartment that does not communicate freely with the plasma. Studies to address this possibility by measuring the effect of raltegravir intensification on important anatomic compartments, such as gut-associated lymphoid tissue, are ongoing.

In addition to examining the effect of raltegravir intensification on residual viremia, we examined the immunologic effects of this intervention. Because residual viremia might affect immune recovery, perhaps by triggering abnormal T cell activation and increasing activation-induced cell death, we assessed whether raltegravir intensification affected the CD4 cell count. We observed an increase in CD4 cell count during the period of raltegravir intensification. A raltegravir-based regimen was also previously found to be associated with greater CD4 cell count gains than an efavirenz-based regimen in a randomized study conducted in treatment-naïve patients [8]. However, the increased CD4 cell counts during raltegravir intensification in the current study did not reach statistical significance and were not associated with a decrease in T cell activation; therefore, the CD4 cell rise may have been due to chance and should be interpreted cautiously. The change in CD4 cell count may be due to an action of raltegravir in a cellular or anatomic compartment that affects T cell homeostasis but does not influence plasma viremia or level of T cell activation in the blood; however, such a mechanism is speculative.

This study also allowed us to examine the relationship between residual viremia and T cell activation. Persistent viremia might explain the observation that T cell activation remains higher in patients who are receiving therapy and have HIV-1 RNA levels of <50 copies/mL than in uninfected individuals [17]. This persistent immune activation may have important clinical consequences; for example, persistent T cell activation is associated with lower CD4 cell count increases in patients receiving ART [17],[18],[19] and may contribute to accelerated atherosclerosis [20] or premature immunosenescence. If residual viremia is inducing T cell activation, one might expect a correlation between the level of viremia and extent of activation. However, in those participants who had detectable residual viremia, we did not find any associations between HIV-1 RNA level at study entry and markers of T cell activation. In addition, raltegravir intensification did not clearly decrease immune activation in the blood, as measured by the percentage of T cells that express CD38 and HLA-DR.

There are some potential limitations of our study. First, the duration of raltegravir intensification was 12 weeks, which may not have been long enough to see an effect of the agent on very long-lived reservoirs. However, if residual viremia arises from new cycles of infection, as opposed to release of virus from stable reservoirs, we would expect to detect an effect on HIV-1 RNA within the 12-week period of intensification. If there is ongoing HIV-1 replication, one would expect that both short- and long-lived cells would be infected—it seems implausible that new infection would only occur in long-lived cells—and, therefore, blocking infection of short-lived cells by adding raltegravir should reduce plasma viremia within a 12-week period. The absence of such an effect argues that residual plasma viremia does not primarily arise from complete cycles of HIV-1 replication and infection of new cells. A second limitation of our study is that the low HIV-1 RNA levels at study entry limit the ability to detect declines after intensification; as a result, our findings do not completely exclude the possibility that ongoing cycles of HIV-1 replication contribute to residual viremia. However, if adding raltegravir substantially reduced HIV-1 replication and residual viremia, we would expect that a higher proportion of patients would have HIV-1 RNA levels below the detection level of the single copy assay during intensification; we did not see such an effect. Finally, as noted above, we cannot exclude the possibility that there is ongoing viral replication in a compartment or compartments that do not contribute to plasma viremia and do not affect the level of T cell activation in blood. Studies of HIV-1 replication in other compartments, such as the gut-associated lymphoid tissue, are required to exclude this possibility.

This study also has several strengths. First, unlike previous single-arm intensification studies, we performed a double-blind placebo-controlled randomized trial, which limits confounding. Second, this study has a longer period of intensification than a recently published trial that examined the effect of 4 weeks of raltegravir intensification [6]. Third, the sample size of the current study allows us to potentially detect smaller effects of intensification on HIV-1 RNA, CD4 cell counts and immune activation than previous smaller single-arm studies could discern. We were able to exclude, with 95% confidence, a median reduction in HIV-1 RNA during raltegravir intensification of more than 0.5 copies/mL in our population. Finally, the cross-over design of the current study made it possible to conduct within-group comparisons of HIV-1 RNA levels before and after raltegravir intensification; the lack of an effect when the drug was either added or subtracted from the regimen strengthens confidence in our results.

In conclusion, in this randomized, double-blind cross-over study, 12 weeks of raltegravir intensification was not found to reduce low-level plasma viremia in patients on currently recommended ART. This finding argues against the hypothesis that ongoing, complete cycles of viral replication and integration are the main source of residual viremia. These results suggest that treatment intensification with raltegravir is unlikely to lead to eradication of HIV-1 infection, and that new therapeutic strategies to eliminate cellular or anatomic reservoirs will be required to cure HIV-1 infection [4],[21].

## Supporting Information

Text S1 Study protocol.
(0.63 MB DOC)

Text S2 CONSORT checklist.
(0.19 MB DOC)

## Abbreviations

ART: antiretroviral therapy
HLA: human leukocyte antigen
LLQ: lower limit of quantification
NNRTI: non-nucleoside reverse transcriptase inhibitor
NRTI: nucleoside reverse transcriptase inhibitor
PI: protease inhibitor
RT: reverse transcriptase
SCA: single copy assay
