# Correction: The Effect of Raltegravir Intensification on Low-level Residual Viremia in HIV-Infected Patients on Antiretroviral Therapy: A Randomized Controlled Trial

**DOI:** 10.1371/annotation/fcb608e3-54c2-4cf8-b1d9-34e86196013f

**Published:** 2011-08-04

**Authors:** Rajesh T. Gandhi, Lu Zheng, Ronald J. Bosch, Ellen S. Chan, David M. Margolis, Sarah Read, Beatrice Kallungal, Sarah Palmer, Kathy Medvik, Michael M. Lederman, Nadia Alatrakchi, Jeffrey M. Jacobson, Ann Wiegand, Mary Kearney, John M. Coffin, John W. Mellors, Joseph J. Eron, on behalf of the AIDS Clinical Trials Group A5244 team

The Academic Editor for this paper was inadvertently omitted. The AE for this paper was Clifford Lane, National Institutes of Health, United States of America. 

