# Supplementary material for: The Effect of Raltegravir Intensification on Low-level Residual Viremia in HIV-Infected Patients on Antiretroviral Therapy: A Randomized Controlled Trial
Source: PLoS Med. 2010 Aug 10;7(8):e1000321. doi: 10.1371/journal.pmed.1000321 (PMC2919424; doi:10.1371/journal.pmed.1000321)
Supplement: Text S1 — Study protocol. (0.63 MB DOC) [file pmed.1000321.s001.doc]

**A5244**

**A Double-Blind, Randomized, Pilot Study to Measure the Effect of Treatment Intensification with a Potent Integrase Inhibitor, Raltegravir (MK-0518), on the Level of Persistent Plasma Viremia below 50 copies/mL in Subjects on Protease Inhibitor- or Non-Nucleoside Reverse Transcriptase Inhibitor-Containing Regimens**

**A Multicenter Trial of the AIDS Clinical Trials Group (ACTG)**

**Sponsored by:**

**The National Institute of Allergy**

**and Infectious Diseases**

**Pharmaceutical Support Provided by:**

**Merck and Company**

**IND #**

**The ACTG Translational Research and**

**Drug Development (TRADD) Scientific**

**Committee: Cara Wilson, M.D., Chair**

**Protocol Chair: Rajesh T. Gandhi, M.D.**

**Protocol Co-Vice Chairs: Joseph Eron, M.D.**

**John Mellors, M.D.**

**DAIDS Clinical Representatives: Sarah Read, M.D.**

**Carla Pettinelli, M.D., Ph.D.**

**Clinical Trials Specialist: Beatrice Kallungal, B.S.**

**FINAL Version 1.0**


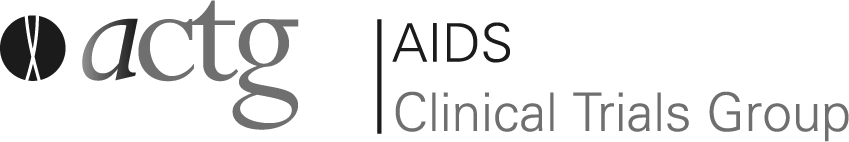
**July 16, 2007**

CONTENTS

Page

SITES PARTICIPATING IN THE STUDY [4](#__RefHeading___Toc172343917)

PROTOCOL TEAM ROSTER [5](#__RefHeading___Toc172343918)

STUDY MANAGEMENT [8](#__RefHeading___Toc172343919)

ACRONYMS [10](#__RefHeading___Toc172343920)

SCHEMA [12](#__RefHeading___Toc172343921)

1.0 HYPOTHESIS AND STUDY OBJECTIVES [13](#__RefHeading___Toc172343922)

1.1 Hypothesis [13](#__RefHeading___Toc172343923)

1.2 Primary Objective [13](#__RefHeading___Toc172343924)

1.3 Secondary Objectives [13](#__RefHeading___Toc172343925)

2.0 INTRODUCTION [13](#__RefHeading___Toc172343926)

2.1 Background [13](#__RefHeading___Toc172343927)

2.2 Rationale [17](#__RefHeading___Toc172343928)

3.0 STUDY DESIGN [18](#__RefHeading___Toc172343929)

4.0 SELECTION AND ENROLLMENT OF SUBJECTS [19](#__RefHeading___Toc172343930)

4.1 Inclusion Criteria [19](#__RefHeading___Toc172343931)

4.2 Exclusion Criteria [20](#__RefHeading___Toc172343932)

4.3 Study Enrollment Procedures [21](#__RefHeading___Toc172343933)

4.4 Coenrollment Guidelines [22](#__RefHeading___Toc172343934)

5.0 STUDY TREATMENT [22](#__RefHeading___Toc172343935)

5.1 Regimens, Administration, and Duration [22](#__RefHeading___Toc172343936)

5.2 Study Product Formulation and Preparation [23](#__RefHeading___Toc172343937)

5.3 Pharmacy: Product Acquisition, Distribution, and Accountability [23](#__RefHeading___Toc172343938)

5.4 Concomitant Medications [23](#__RefHeading___Toc172343939)

5.5 Adherence Assessment [26](#__RefHeading___Toc172343940)

6.0 CLINICAL AND LABORATORY EVALUATIONS [27](#__RefHeading___Toc172343941)

6.1 Schedule of Events [27](#__RefHeading___Toc172343942)

6.2 Timing of Evaluations [29](#__RefHeading___Toc172343943)

6.3 Instructions for Evaluations [31](#__RefHeading___Toc172343944)

7.0 CLINICAL MANAGEMENT ISSUES [36](#__RefHeading___Toc172343945)

7.1 Toxicity Management [36](#__RefHeading___Toc172343946)

8.0 CRITERIA FOR DISCONTINUATION [40](#__RefHeading___Toc172343947)

8.1 Permanent Study Drug Discontinuation [40](#__RefHeading___Toc172343948)

8.2 Premature Study Discontinuation [40](#__RefHeading___Toc172343949)

9.0 STATISTICAL CONSIDERATIONS [40](#__RefHeading___Toc172343950)

9.1 General Design Issues [40](#__RefHeading___Toc172343951)

9.2 Endpoints [40](#__RefHeading___Toc172343952)

9.3 Randomization and Stratification [41](#__RefHeading___Toc172343953)

9.4 Sample Size and Accrual [41](#__RefHeading___Toc172343954)

9.5 Monitoring [42](#__RefHeading___Toc172343955)

9.6 Analyses [42](#__RefHeading___Toc172343956)

10.0 PHARMACOLOGY PLAN [44](#__RefHeading___Toc172343957)

11.0 DATA COLLECTION AND MONITORING AND ADVERSE EVENT REPORTING [44](#__RefHeading___Toc172343958)

11.1 Records to Be Kept [44](#__RefHeading___Toc172343959)

11.2 Role of Data Management [44](#__RefHeading___Toc172343960)

11.3 Clinical Site Monitoring and Record Availability [44](#__RefHeading___Toc172343961)

11.4 Expedited Adverse Event Reporting to DAIDS [45](#__RefHeading___Toc172343962)

12.0 HUMAN SUBJECTS [45](#__RefHeading___Toc172343963)

12.1 Institutional Review Board (IRB) Review and Informed Consent [45](#__RefHeading___Toc172343964)

12.2 Subject Confidentiality [46](#__RefHeading___Toc172343965)

12.3 Study Discontinuation [46](#__RefHeading___Toc172343966)

13.0 PUBLICATION OF RESEARCH FINDINGS [46](#__RefHeading___Toc172343967)

14.0 BIOHAZARD CONTAINMENT [46](#__RefHeading___Toc172343968)

15.0 REFERENCES [47](#__RefHeading___Toc172343969)

APPENDIX I: SPECIMEN COLLECTION, PROCESSING, AND SHIPMENT [49](#__RefHeading___Toc172343970)

APPENDIX II: SAMPLE INFORMED CONSENT

###### SITES PARTICIPATING IN THE STUDY

This study is open to all U.S. clinical trials units (CTUs) and their clinical research sites (CRSs).

###### PROTOCOL TEAM ROSTER

Chair

Rajesh T. Gandhi, M.D.

Massachusetts General Hospital

GRJ 504

55 Fruit Street

Boston, MA 02114

Phone: (617) 724-9690

FAX: (617) 726-7653

E-Mail: [rgandhi@partners.org](mailto:rgandhi@partners.org)

Co-Vice Chair

Joseph J. Eron Jr., M.D.

University of North Carolina at Chapel Hill

130 Mason Farm Road

Bioinformatics Building

Campus Box 7215

Chapel Hill, NC 27599-7215

Phone: (919) 843-2722

FAX: (919) 966-8928

E-Mail: [jeron@med.unc.edu](mailto:jeron@med.unc.edu)

Co-Vice Chair/Co-Virologist

John W. Mellors, M.D.

University of Pittsburgh Medical Center

Scaife Hall, Suite 818

3550 Terrace Street

Pittsburgh, PA 15261

Phone: (412) 624-8512

FAX: (412) 383-7982

E-Mail: [mellors@dom.pitt.edu](mailto:mellors@dom.pitt.edu)

DAIDS Clinical Representatives

Carla Pettinelli, M.D., Ph.D.

HIV Research Branch

TRP, DAIDS, NIAID, NIH

6700-B Rockledge Drive, MSC 7624

Bethesda, MD 20892-7624

Phone: (301) 402-5582

FAX: (301) 435-9282

E-Mail: [**cpettinell@niaid.nih.gov**](mailto:cpettinell@niaid.nih.gov)

DAIDS Clinical Representatives (Cont’d)

Sarah Read, M.D.

HIV Research Branch

TRP, DAIDS, NIAID, NIH

6700-B Rockledge Drive

Bethesda, MD 20892-7624

Phone: (301) 451-2757

FAX: (301) 435-9282

E-Mail: [readsa@niaid.nih.gov](mailto:readsa@niaid.nih.gov)

Clinical Trials Specialist

Beatrice Kallungal, B.S.

ACTG Operations Center

Social & Scientific Systems

8757 Georgia Avenue, 12th Floor

Silver Spring, MD 20910-3714

Phone: (301) 628-3000

FAX: (301) 628-3302

E-Mail: [bkallungal@s-3.com](mailto:bkallungal@s-3.com)

Statisticians

Ellen Chan, M.Sc.

SDAC/Harvard School of Public Health

FXB Building, Room 539

651 Huntington Avenue

Boston, MA 02115

Phone: (617) 432-4879

FAX: (617) 432-3163

E-Mail: [echan@sdac.harvard.edu](mailto:echan@sdac.harvard.edu)

Lu (Summer) Zheng, Ph.D.

SDAC/Harvard School of Public Health

FXB Building, Room 613

651 Huntington Avenue

Boston, MA 02115-6017

Phone: (617) 432-3021

FAX: (617) 432-2843

E-Mail: [szheng@sdac.harvard.edu](mailto:szheng@sdac.harvard.edu)

Data Manager

Jennifer Janik, M.S.

Frontier Science & Technology Research Foundation

4033 Maple Road

Amherst, NY 14226-1056

Phone: (716) 834-0900 x7287

FAX: (716) 834-8432

E-Mail: [janik.jennifer@fstrf.org](mailto:janik.jennifer@fstrf.org)

DAIDS Pharmacist

Ana Martinez, R.Ph.
Pharmaceutical Affairs Branch
DAIDS, NIAID, NIH

Room 5115
6700-B Rockledge Drive, MSC 7620
Bethesda, MD 20892-7620
Phone: (301) 435-3734
FAX: (301) 402-1506

E-Mail: [amartinez@niaid.nih.gov](mailto:amartinez@niaid.nih.gov)

Immunologist

Jeffrey M. Jacobson, M.D.

Drexel University College of Medicine

245 N. 15th Street, MS461

Philadelphia, PA 19102

Phone: (215) 762-6555

E-Mail: [jeffrey.jacobson@drexelmed.edu](mailto:jeffrey.jacobson@drexelmed.edu)

Investigator/Co-Virologist

Sarah Palmer, Ph.D.

Virology Core Facility

National Cancer Institute at Frederick

1050 Boyles Street, Bldg. 535, Rm. 108D

Frederick, MD 21702-1201

Phone: (301) 846-5599

FAX: (301) 846-6013

E-Mail: [spalmer@ncifcrf.gov](mailto:spalmer@ncifcrf.gov)

Investigators

Ronald J. Bosch, Ph.D.

Statistical & Data Analysis Center

Harvard School of Public Health

FXB Building, Room 603

651 Huntington Avenue,

Boston, MA 02115-6017

Phone: (617) 432-3024

FAX: (617) 432-2843

E-Mail: [ronbosch@sdac.harvard.edu](mailto:ronbosch@sdac.harvard.edu)

Richard T. D’Aquila, M.D.

Vanderbilt University Medical Center

A-2200 Medical Center North

1161 21st Avenue South

Nashville, TN 37232-2582

Phone: (615) 322-8972

FAX: (615) 322-3171

E-Mail: [richard.daquila@vanderbilt.edu](mailto:richard.daquila@vanderbilt.edu)

Lisa M. Demeter, M.D.

University of Rochester Medical Center

601 Elmwood Avenue

Box 689

Rochester, NY 14642-0001

Phone: (585) 275-4764

FAX: (585) 442-9328

E-Mail: [lisa_demeter@urmc.rochester.edu](mailto:lisa_demeter@urmc.rochester.edu)

David M. Margolis, M.D.

University of North Carolina at Chapel Hill

Michael Hooker Research Center, CB#7435

Chapel Hill, NC 27599-7435

Phone: (919) 966-6388

FAX: (919) 966-2089

E-Mail: [dmargo@med.unc.edu](mailto:dmargo@med.unc.edu)

Field Representative

Barbara Philpotts, R.N., B.S.N.

Case Western Reserve University

Room 301C, Foley Building

2061 Cornell Road

Cleveland, OH 44106

Phone: (216) 844-2738

FAX: (216) 844-5523

E-Mail: [philpotts.barbara@clevelandactu.org](mailto:philpotts.barbara@clevelandactu.org)

Laboratory Technologist

Betty A. Donoval

Department of Immunology

Rush Medical Center

1735 W. Harrison, 641 Cohn

Chicago, IL 60612

Phone: (312) 942-2212

FAX: (312) 942-5206

E-Mail: [betty_donoval@rsh.net](mailto:betty_donoval@rsh.net)

NCAB Representative

Robert Levaro
1418 E. Via Soledad
Tucson, AZ 85718
Phone: (520) 232-1265
E-Mail: [rlevaro@comcast.net](mailto:rlevaro@comcast.net)

Industry Representative

Randi Leavitt, M.D.

Merck and Company

Mail stop UG3D-30

351 N. Sumneytown Pike

North Wales, PA 19454

Phone: (267) 305-7518

FAX: (267) 305-6530

E-Mail: [randi_leavitt@merck.com](mailto:randi_leavitt@merck.com)

Consultant

John Coffin, M.D.

Department of Molecular Biology

Tufts University

136 Harrison Avenue

Boston, MA 02111-1817

Phone: (617) 636-6528

FAX: (617) 636-8086

E-Mail: [jcoffin_par@opal.tufts.edu](mailto:jcoffin_par@opal.tufts.edu)

Laboratory Data Coordinator

Heather Sprenger, M.S.

Frontier Science and Technology Research Foundation Inc.

4033 Maple Road

Amherst, NY 14226-1056

Phone: (716) 834-0900 x7262

FAX: (716) 833-0655

E-Mail: [sprenger.heather@fstrf.org](mailto:sprenger.heather@fstrf.org)

###### STUDY MANAGEMENT

All questions concerning this protocol should be sent to [actg.teamA5244@fstrf.org](mailto:actg.teamA5244@fstrf.org) via e-mail. The appropriate team member will respond with a "cc" to [actg.teamA5244@fstrf.org](mailto:actg.teamA5244@fstrf.org). A response should generally be received within 24 hours (Monday-Friday).

Protocol E-mail Group

Sites registering to this study should contact the Computer Support Group at the Data Management Center via e-mail ([actg.user.support@fstrf.org](mailto:actg.user.support@fstrf.org)) (include the protocol number in the subject line) to have the relevant personnel at the site added to the actg.protA5244 e-mail group as soon as possible. Inclusion in the protocol e-mail group will ensure that sites receive important information about the study during its implementation and conduct.

Clinical Management

For questions concerning entry criteria, toxicity management, concomitant medications, and coenrollment, contact the protocol Chair/Co-Vice Chairs. Send an e-mail message to [**actg.teamA5244@fstrf.org**](mailto:actg.teamA5244@fstrf.org) (ATTN: Rajesh Gandhi, M.D., Joseph Eron, M.D., and John Mellors, M.D.). Include the protocol number (A5244), patient identification number (PID), and a brief relevant history.

Laboratory

For questions specifically related to immunologic or virologic laboratory tests, contact the protocol Immunologist or Co-Virologists. Send an e-mail message to [actg.teamA5244@fstrf.org](mailto:actg.teamA5244@fstrf.org) (ATTN: Jeffrey Jacobson, M.D. or John Mellors, M.D./Sarah Palmer, Ph.D.).

Data Management

For nonclinical questions about inclusion/exclusion criteria, case report forms (CRF), the CRF schedule of events, randomization/registration, transfers, delinquencies, and other data management issues, contact the Data Manager. Send an e-mail message to [actg.teamA5244@fstrf.org](mailto:actg.teamA5244@fstrf.org) (ATTN: Jennifer Janik, M.S.). Include the protocol number (A5244), PID, and a detailed question.

Randomization

For randomization questions or problems and SID (study identification number) lists, contact the SDAC/DMC (Statistical and Data Analysis Center /Data Management Center) programmers. Call the SDAC/DMC Randomization Desk at (716) 898-7301 or e-mail [sdac.random.desk@fstrf.org.](mailto:actg.user.support@fstrf.org)

Computer and Screen Problems

Contact the SDAC/DMC programmers. Send a message to [actg.user.support@fstrf.org](mailto:actg.user.support@fstrf.org) via e-mail or call (716) 834-0900 x7302.

Protocol Document Questions

For questions concerning the protocol document, contact the Clinical Trials Specialist. Send an e-mail message to [actg.teamA5244@fstrf.org](mailto:actg.teamA5244@fstrf.org) (ATTN: Beatrice Kallungal, B.S.).

Copies of the Protocol

To request hard copies of the protocol, send a message to [ADULT.OPS@fstrf.org](mailto:ACTG.OPS@fstrf.org) (ATTN: Diane Delgado) via e-mail. Electronic copies can be downloaded from the Members area of the ACTG Web site ([http://aactg.s-3.com](http://aactg.s-3.com/)).

Product Package Inserts or Investigator Brochures

To request copies of product package inserts or investigator brochures contact the DAIDS Regulatory Compliance Center (RCC) at [RIC@tech-res.com](mailto:RIC@tech-res.com) or call (301) 897-1708.

Protocol Registration

Send an e-mail message to [Protocol@tech-res.com](../Protocol@tech-res.com) or call (301) 897-1707.

Study Drug

For questions or problems regarding study drug, dose, supplies, records, and returns, call Ana Martinez, R.Ph., Protocol Pharmacist, at (301) 496-8213.

IND Number or Questions

Contact the DAIDS RCC at [Regulatory@tech-res.com](../Protocol@tech-res.com) or call (301) 897-1706.

Study Drug Orders

Call the Clinical Research Products Management Center at (301) 294-0741.

Expedited Adverse Event (EAE) Reporting/Questions

Contact DAIDS through the RCC Safety Office at [**RCCSafetyOffice**](../ProtShell.doc)[**@tech-res.com**](../SafetyOffice@tech-res.com) or call 1-800-537-9979 or 301-897-1709; or fax 1-800-275-7619 or 301-897-1710.

Phone Calls

Any phone calls must be documented by e-mail to [**actg.teamA5244@fstrf.org**](mailto:actg.teamA5244@fstrf.org). This will be the site’s responsibility.

Protocol-Specific Web Page

Additional information concerning study management of ACTG studies can be found on the A5244 ACTG Web page at [*http://aactg.s-3.com/members/ps/5244/ps5244.htm*](http://aactg.s-3.com/members/ps/5244/ps5244.htm).

###### ACRONYMS

3TC lamivudine (Epivir®)

ACTG AIDS Clinical Trials Group

AE adverse event/experience

AIDS acquired immunodeficiency syndrome

ALT alanine aminotransferase (SGPT)

ANC absolute neutrophil count

ART antiretroviral therapy

AST aspartate aminotransferase (SGOT)

β-HCG beta-human chorionic gonadotropin

BID twice daily

BRI Biomedical Research Institute (specimen repository)

CK creatine kinase

CLIA Clinical Laboratory Improvement Amendments

CRF case report form

CRPMC Clinical Research Products Management Center

CRS Clinical Research Site

CTU Clinical Trials Unit

DAIDS Division of AIDS

DMC Data Management Center

EAE expedited adverse event

EFV efavirenz (Sustiva®)

ELISA enzyme-linked immunosorbent assay

FDA Food and Drug Administration

FSTRF Frontier Science and Technology Research Foundation, Inc.

HAART highly active antiretroviral therapy

HLA human leukocyte antigen

IATA International Air Transport Association

IC95 Concentration that results in 95% inhibition

IND investigational new drug

IQA Immunology Quality Assurance (IQA)

IRB institutional review board

LDMS Laboratory Data Management System

NIAID National Institute of Allergy and Infectious Diseases

NIH National Institutes of Health

NNRTI non-nucleoside analogue reverse transcriptase inhibitor

NRTI nucleoside analogue reverse transcriptase inhibitor

OHRP Office for Human Research Protections (formerly OPRR)

PAB Pharmaceutical Affairs Branch

PBMC peripheral blood mononuclear cell

PCR polymerase chain reaction

PI protease inhibitor

PID patient identification number

PK pharmacokinetics

PO per os (by mouth)

RCC Regulatory Compliance Center

RT reverse transcriptase

RTV ritonavir

SAE serious adverse event

SCA single copy assay

SDAC Statistical and Data Analysis Center

SGOT serum glutamic-oxaloacetic transaminase (AST)

SGPT serum glutamic-pyruvic transaminase (ALT)

SIC sample informed consent

SID study identification number (refers to prescriptions written on study)

SMC Safety Monitoring Committee

SOE schedule of events

TRP Therapeutics Research Program

ULN upper limit of normal

WBC white blood cell

###### SCHEMA

A5244

A Double-Blind, Randomized, Pilot Study to Measure the Effect of Treatment Intensification with a Potent Integrase Inhibitor, Raltegravir (MK-0518), on the Level of Persistent Plasma Viremia below 50 copies/mL in Subjects on Protease Inhibitor- or Non-Nucleoside Reverse Transcriptase Inhibitor-Containing Regimens

DESIGN A5244 is a double-blind, randomized, placebo-controlled cross-over pilot study to measure the effect of treatment intensification with a potent integrase inhibitor, raltegravir (MK-0518), on the level of persistent plasma HIV-1 RNA level <50 copies/mL in subjects on PI- or NNRTI-containing regimens.

DURATION 24 weeks.

SAMPLE SIZE 50 subjects.

POPULATION HIV-1 infected men and women on PI- or NNRTI-containing antiretroviral therapy (ART) for at least 12 months before study entry with plasma HIV-1 RNA <50 copies/mL and CD4+ T-cell count ≥200 cells/mm^3^.

STRATIFICATION Subjects will be stratified by whether they are on a PI- or NNRTI-containing regimen.

REGIMEN At study entry the subjects will be randomized to one of the following arms:

Arm A (immediate intensification): At week 0, continue entry regimen (at least 2 NRTIs + PI or NNRTI [not provided by the study]) and add raltegravir for 12 weeks. At week 12, stop raltegravir and add placebo for 12 weeks.

Arm B (delayed intensification): At week 0, continue entry regimen (at least 2 NRTIs + PI or NNRTI [not provided by the study]) and add placebo for 12 weeks. At week 12, stop placebo and add raltegravir for 12 weeks.

**Arm B**

**Placebo**

**Raltegravir**

**Wk 0**

**Wk 12**

**Wk 24**

**Placebo**

**Raltegravir**

**Arm A**

**Randomized**

# 1.0 HYPOTHESIS AND STUDY OBJECTIVES

## 1.1 Hypothesis

The level of plasma HIV-1 RNA on real-time PCR single copy assay (SCA) will be lower in subjects on a stable PI- or NNRTI-containing regimen after the addition of raltegravir (MK-0518), compared with subjects who do not add raltegravir, reflecting suppression of ongoing low-level viral replication in subjects receiving raltegravir.

## 1.2 Primary Objective

To compare the HIV-1 RNA level on SCA averaged between weeks 10 and 12 (10/12) in subjects who add raltegravir to a PI- or NNRTI-containing regimen to the level in subjects who do not add raltegravir to a PI- or NNRTI-containing regimen.

## 1.3 Secondary Objectives

1.3.1 To compare the within-subject difference in HIV-1 RNA level by SCA from baseline to the level at weeks 10/12 between the arms. The change in HIV-1 RNA level from baseline to weeks 10/12 will be compared to the change from weeks 10/12 to weeks 22/24 within each treatment arm.

1.3.2 To evaluate the effect on CD4+ T-cell counts of adding raltegravir to a PI- or NNRTI-containing regimen.

1.3.3 To evaluate the effect on CD8+ T-cell counts of adding raltegravir to a PI- or NNRTI-containing regimen.

- - 1. To evaluate the effect on CD4+ T-cell activation of adding raltegravir to a PI- or NNRTI-containing regimen.
    2. To evaluate the effect on CD8+ T-cell activation of adding raltegravir to a PI- or NNRTI-containing regimen.
    3. To evaluate safety and tolerability of adding raltegravir to a PI- or NNRTI-containing regimen.

# 2.0 INTRODUCTION

## 2.1 Background

Although ART has greatly reduced the morbidity and mortality from HIV-1 infection, current treatment does not eradicate the infection. A combination of medications that block the viral enzymes reverse transcriptase (RT) and protease (PR) can suppress the plasma HIV-1 RNA to ≤50 copies/mL, but most individuals who stop ART have a rapid rebound in plasma viremia. One likely explanation for this failure to eradicate HIV-1 is the persistence of the virus in long-lived reservoirs. In subjects on current classes of ART (i.e., RT and PR inhibitors), the decay rate of HIV-1 in latently infected resting memory CD4+ T-cells is estimated to be >44 months, which means more than 70 years of therapy would be needed to eradicate the virus^(1;2)^.

This long half-life of the latent reservoir may be due to ongoing low levels of viral replication in patients on standard therapy with RT and PR inhibitors. In fact, recent data indicate that the majority of infected individuals on current therapies have persistent HIV-1 RNA levels below the current limit of detection of commercially available assays when measured by more sensitive research tests, such as a real-time PCR SCA^(3)^. This internally controlled real-time RT-initiated PCR assay quantifies HIV-1 RNA concentration down to 1 copy/mL of plasma. Using this assay, testing of plasma samples from 15 subjects who were receiving ART and who had <75 copies/mL of HIV-1 RNA revealed persistent viremia in all 15 subjects, with HIV-1 RNA levels ranging from 1 to 32 copies/mL (median, 13 copies/mL)^(4)^. If this low level viremia reflects ongoing, complete cycles of viral replication, this may replenish the latent reservoir and slow its decay. In this case, intensification with drugs that more completely block viral replication may reduce the level of persistent viremia, decrease the number of replication-competent HIV proviruses in CD4+ T cells, and lead to more rapid decay of the latent reservoir.

Since the latent reservoir is generated by integration of HIV-1 into the host genome, an inhibitor of viral integration may be particularly effective at reducing the number of cells in the latent reservoir. Recently a new integrase inhibitor, raltegravir, has been tested in phase II clinical trials, and has been found to be safe and highly effective in reducing plasma HIV-1 RNA levels^(5)^. Thus, addition of this potent integrase inhibitor to PI or NNRTI-containing ART may lead to more complete blockade of viral replication, resulting in a reduction in persistent plasma viremia, as well as potentially a decreased provirus copy number in CD4+ T cells and potentially more rapid decay of the latent reservoir.

Raltegravir

Raltegravir (MK-0518) is an HIV-1 integrase inhibitor in development by Merck and Company. Raltegravir has potent in vitro activity against HIV-1, with an IC95 of 33 nM in 50% human serum. The drug is primarily metabolized by glucuronidation and is not a known CYP450 inducer, inhibitor, or substrate. Studies suggest that the dose of raltegravir does not need to be adjusted when given with other antiretroviral agents. For example, interactions between ritonavir (RTV) or efavirenz (EFV) and raltegravir were studied in 2 placebo-controlled studies, each with 12 young, healthy male subjects^(6)^. Raltegravir levels were not substantially affected when subjects received concomitant RTV. In the presence of EFV, raltegravir levels were modestly reduced (area under the curve was decreased by 36%, the trough concentration by 21%, and the maximum concentration by 36%). There was not a substantial effect on raltegravir t_max_ or t_½_ in the presence or absence of EFV.  Given the overall PK parameters in the presence or absence of EFV, the interaction is unlikely to be clinically meaningful. Coadministration of raltegravir with either RTV or EFV was generally well-tolerated in these subjects.

Raltegravir has demonstrated potent in vivo activity in treatment-experienced and treatment-naïve HIV-infected subjects. In 167 treatment-experienced subjects^(7)^, raltegravir, at one of three doses (200 mg, 400 mg, and 600 mg, each given BID) lowered viral load by at least 2 log_10_ copies/mL in all subjects within 2 weeks after initiating treatment and suppressed viral load to less than 50 copies/mL in 56-72% of subjects within 16 weeks. 50% of those entering the trial had a baseline phenotypic susceptibility score of 0 for drugs used to design the background regimen (other than T20 and raltegravir), indicating a high degree of antiretroviral drug resistance. 38% used enfuvirtide in the background regimen. Adverse event (AE) rates in subjects receiving raltegravir were comparable to controls (Table 1). Headache, fatigue, and dizziness were the most commonly reported AEs.

In a trial of treatment-naïve subjects, 197 subjects were randomized to receive one of 4 doses of raltegravir or EFV with tenofovir and emtricitabine. At week 24, 85-95% of subjects in all dose groups of raltegravir achieved an HIV-1 RNA level of <50 copies/mL. The HIV-1 RNA level declined more rapidly in subjects who received raltegravir than in those who received EFV. Raltegravir was generally well-tolerated (Table 2), with only one subject discontinuing drug due to elevated AST^(8)^. The dose of 400 mg BID was chosen for further development of raltegravir. In the studies of raltegravir in both treatment-naïve and treatment-experienced subjects each dose arm had similar efficacy. The 400 mg BID dose was chosen for phase III development to ensure optimal drug concentrations even in the face of modest drug interactions, such as described above with EFV, but also to allow for a margin of safety for long-term administration.

In two phase III trials (BENCHMRK-1 and -2)^(9;10)^, raltegravir was compared to placebo each with optimized background therapy in patients failing ART with triple-class resistant HIV. At week 16, subjects receiving raltegravir had significantly higher rates of HIV-1 RNA levels <50 copies/mL (61% in BENCHMRK-1 and 62% in BENCHMRK-2) compared with subjects receiving placebo (33% and 36%). In both studies, subjects receiving raltegravir had significantly greater increases in CD4+ cell count at week 16 than subjects receiving placebo. In both trials, raltegravir was generally well-tolerated, with an AE profile similar to that of placebo.

Table 1: Protocol 005: Most Common Drug-Related Clinical Adverse Events

(Incidence ≥5% in at least one treatment group)

|  | Raltegravir* | | | Placebo* |
| --- | --- | --- | --- | --- |
|  | 200 mg  N = 43 | 400 mg  N = 45 | 600 mg  N = 45 | N = 45 |
| Diarrhea | 4 (9%) | 1 (2%) | 0 (0%) | 7 (16%) |
| Nausea | 3 (7%) | 2 (4%) | 5 (11%) | 5 (11%) |
| Fatigue | 4 (9%) | 0 (0%) | 2 (4%) | 1 (2%) |
| Injection site reaction | 1 (2%) | 3 (7%) | 5 (11%) | 3 (7%) |
| Headache | 4 (9%) | 0 (0%) | 2 (4%) | 3 (7%) |
| Pruritus | 1 (2%) | 2 (4%) | 3 (7%) | 0 (0%) |

* Given BID with optimized background therapy (OBT)^(11)^

Table 2: Protocol 004: Common (≥5%) Drug-Related Adverse Events

|  | MK 0518* (all doses) N=160  (%) | Efavirenz*  N=38  (%) |
| --- | --- | --- |
| Nausea | 11 (7%) | 13 (34%) |
| Headache | 9 (6%) | 24 (63%) |
| Dizziness | 8 (5%) | 26 (68%) |
| Diarrhea | 7 (4%) | 11 (29%) |
| Insomnia | 7 (4%) | 11 (29%) |
| Abnormal dreams | 6 (4%) | 18 (47%) |
| Flatulence | 6 (4%) | - (0%) |

* With tenofovir and lamivudine

These studies suggest that raltegravir is a potent and well-tolerated antiviral agent. Please refer to the raltegravir investigator’s brochure for additional details regarding this drug.

Deaths

Thirteen deaths have been reported in participants on trial: 8 in participants on raltegravir during the double-blinded phase, 3 in participants on placebo during the double-blinded phase, and 2 on Raltegravir during the open-label phase. The raltegravir arms had more participants and longer follow-up than the placebo phase, thus the adjusted incidence of death was 1.568 per 100 patient-years on raltegravir and 1.771 per 100 patient years on placebo.

The deaths while on raltegravir were: 4 due to infectious diseases (cryptococcal meningitis, bronchopulmonary aspergillosis and pulmonary tuberculosis, bronchopneumonia with rectal hemorrhage and septic shock, and sepsis with bradycardia and shock), 3 due to cancer and complications, 1 due to suicide, 1 due to myocardial infarction, and 1 due to progressive multifocal leukencephalopathy. The deaths in the placebo group were due to urosepsis, pneumonia, and mycobacterium avium complex.

Neoplasms

In the double blind portions of the studies, 10 neoplasms occurred in participants on raltegravir and 1 in a patient on a comparator arm. The double blind portion of studies provides an opportunity to evaluate the risk in patients receiving raltegravir as well as non-raltegravir containing regimens. It should be noted that the cumulative time at risk (duration of follow-up) for patients receiving raltegravir is not balanced compared to comparator arms because of study design (3:1 or 4:1 randomization ratios in Phase 2 studies and 2:1 in Phase 3 studies). The time at risk for the raltegravir arms as of frozen file for Protocols 004, 005, 018, and 019 was 508 patient years. For the comparator arms, the time at risk was 169 patient years. Adjustment using patient-years is necessary to address the imbalance in time at risk. The adjusted rates of neoplasms per 100 patient-years in the double blind portions of the studies are 1.970 in the raltegravir arms and 0.592 in the comparator groups. This constitutes a RR of 3.328 (95% confidence interval 0.47, 144.45). Based on these data, no specific cancer risk attributable to raltegravir is apparent.

However, the imbalance in neoplasms prompted a more detailed review of all available data. This review encompassed the double blind phases of Protocols 004, 005, 018, and 019, the open label arms of Protocols 005, 018, and 019, the ongoing blinded study Protocol 021, and the expanded access environment.

Overall, a total of 21 neoplasms were reported as of January 22, 2007. Of the 21 neoplasms, 17 participants with 18 neoplasms were in raltegravir groups, 1 neoplasm in the comparator arms and 2 neoplasms during the screening period prior to treatment. The 18 malignancies occurring on raltegravir during the treatment periods were: 2 Kaposi’s sarcoma, 6 lymphomas (2 Hodgkin’s disease and 4 non-Hodgkin’s lymphoma), 4 squamous cell carcinomas (1 rectal, 1 laryngeal, 2 skin), 3 anal cancers, 1 basal cell, 1 hepatocellular carcinoma, and 1 rectal cancer. Four (4) of the cancers were recurrent, and most were associated with well known risk factors such as AIDS, oncogenic viruses (papillomavirus infection, hepatitis B virus infection), and tobacco, and some had suggestive symptoms and/or signs present prior to or at the time of enrollment. Most of the cancers were detected within a short period after enrollment (3 months), and several showed advanced disease state at the time of diagnosis, suggesting the malignancies might have been present, but not detected at the time of enrollment.

Though calculation of relative risk is only possible in double blind studies with comparator arms, the open label arms in the raltegravir clinical development program afforded the opportunity to evaluate the rates of neoplasm in a population with a longer total time at risk. The number of patient years of follow-up for Protocols 004, 005, 018, and 019 including the double blind portions and the open label portions was 619 patient years for the raltegravir arm, constituting an additional 111 patient years of follow-up as compared to the double blind portions only. With this additional follow-up, the case rate per 100 patient-years was stable at 1.940, indicating that the patient year adjusted rate for raltegravir seen in the earlier portions of the study did not increase with additional follow-up for patients on raltegravir.

In summary, while there is an apparent imbalance in the rates of malignancies reported for the raltegravir groups and the comparator groups, there does not appear to be any direct evidence of drug relationship to these events. The study population in which most of these events occurred has highly advanced immunodeficiency and the rates of malignancies observed are within the expected rates for patients with advanced infection. Furthermore, a variety of cancers was reported, and the specific types were expected in this population. Additionally, most were identified soon after study entry, and several were recurrent, suggesting these were likely to be present at or before the time of study entry. Finally, the rate of malignancies does not appear to increase with additional follow-up in patients on raltegravir.

## 2.2 Rationale

To test the hypothesis that ART intensification with an integrase inhibitor will reduce persistent viremia, we propose to conduct a pilot trial to measure the effect on HIV-1 RNA levels of adding raltegravir to the regimen of subjects who have plasma HIV-1 RNA levels <50 copies/mL on PI- or NNRTI-containing therapy.

# 3.0 STUDY DESIGN

A5244 is a double-blind, randomized, placebo-controlled cross-over pilot study to measure the effect of treatment intensification with a potent integrase inhibitor, raltegravir, on the level of persistent plasma HIV-1 RNA level <50 copies/mL in subjects on PI- or NNRTI-containing regimens.

HIV-1-infected subjects on a PI- or NNRTI-containing ART regimen for at least 12 months who have had HIV-1 RNA <50 copies/mL for at least 6 months and have detectable virus by SCA will be randomized 1:1 to add either raltegravir (Arm A, immediate intensification) or placebo (Arm B, delayed intensification) for 12 weeks. At week 12, subjects will cross-over and switch study drug (raltegravir or placebo), while continuing their entry regimen: that is, at week 12 subjects in Arm A will stop raltegravir and add placebo, while subjects in Arm B will stop placebo and add raltegravir to their entry regimen.

Subjects will have measurements of HIV-1 RNA by SCA, CD4+ and CD8+ T-cell counts, and activated CD4+ and CD8+ T-cell percentage by advanced flow at pre-entry and entry. The average of these measurements will be used to establish their baseline values. Following entry, subjects will have plasma HIV-1 RNA SCA samples drawn at weeks 2, 4, 10, 12, 14, 16, 22, and 24. The HIV-1 RNA SCA values at weeks 10 and 12 and at 22 and 24 will be averaged. Subjects who have met the protocol definition of virologic failure (two consecutive viral loads ≥50 copies/mL) will no longer contribute samples for the SCA.

The primary analysis will be a comparison of the average of weeks 10 and 12 SCA values between subjects in Arms A and B. The difference between the baseline (average of pre-entry and entry) and the average of weeks 10 and 12 SCA values will also be compared between arms. Furthermore, the within-subject effect of intensification will be evaluated by contrasting the slope of SCA values before versus after week 12.

The cross-over design allows assessment of the effect of intensification with raltegravir between the two arms at weeks 10/12. It also provides an opportunity to examine the within-subject effect of intensification in both treatment arms as well as to evaluate whether there is an increase in HIV-1 RNA on SCA after discontinuation of raltegravir by subjects in arm A. Subjects will be stratified by whether they are on a PI- or NNRTI-containing regimen. The stratification limits possible confounding by the background regimen.

# 4.0 SELECTION AND ENROLLMENT OF SUBJECTS

## 4.1 Inclusion Criteria

4.1.1 HIV-1 infection, as documented by any licensed ELISA test kit and confirmed by Western blot at any time prior to study entry. HIV-1 culture, HIV-1 antigen, plasma HIV-1 RNA, or a second antibody test by a method other than ELISA is acceptable as an alternative confirmatory test*.*

4.1.2 ART for at least 12 months prior to study entry with a regimen that includes at least two NRTI and either a NNRTI or a RTV-boosted PI.

4.1.3 No change in ART regimen for at least 3 months prior to study entry.

4.1.4 CD4+ cell count ≥200/mm^3^ at screening (obtained within 60 days prior to study entry) at any laboratory that has a CLIA certification or its equivalent.

4.1.5 Documentation of HIV-1 RNA below the limit of quantification of an ultrasensitive assay (for example, <50 copies/mL on Roche Amplicor HIV-1 Monitor Test, <75 copies/mL on the Versant HIV-1 RNA assay by branched DNA) for a period of at least 6 months before study entry, verified by at least two measurements separated from each other by at least 60 days. The first measurement must be performed at least 6 months prior to study entry, and the second measurement must be performed within 6 months prior to study entry (the measurement obtained at the time of screening may be used to fulfill this criterion). The HIV-1 RNA measurements may be performed by any laboratory that has a CLIA certification or its equivalent.

4.1.6 Screening HIV-1 RNA < 50 copies/mL using Roche Amplicor HIV-1 RNA Ultrasensitive assay obtained within 60 days of entry.

4.1.7 All HIV-1 RNA levels obtained within 6 months prior to study entry are below the limits of quantification on all tests.

4.1.8 Subjects must have documentation in the form of a laboratory report of a pre-ART HIV-1 RNA level of >100,000 copies/mL.

4.1.9 Detectable HIV-1 RNA ≥1 copy on the screening SCA.

4.1.10 Confirmation of the availability of the stored pre-entry plasma sample for HIV-1 RNA SCA determination. The site must confirm that this sample has been entered into the Laboratory Data Management System (LDMS).

4.1.11 Laboratory values obtained within 60 days prior to entry:

- Absolute neutrophil count (ANC) ≥750/mm^3^
- Hemoglobin ≥9.0 g/dL for female subjects and ≥10.0 g/dL for male subjects
- Platelet count ≥50,000/mm^3^
- Calculated creatinine clearance (CrCl) ≥30 mL/min, as estimated by the Cockcroft-Gault equation*

NOTE: *Calculation for the Cockcroft-Gault equation is available at <https://www.fstrf.org/apps/cfmx/apps/common/Portal/index.cfm>

- AST (SGOT), ALT (SGPT), and alkaline phosphatase ≤5 × ULN
- Total bilirubin ≤2.5 × ULN. If the subject is taking an indinavir- or atazanavir-containing regimen at the time of screening, total bilirubin ≤5 x ULN is acceptable.

4.1.12 For females of reproductive potential (women who have not been post-menopausal for at least 24 consecutive months, i.e., who have had menses within the preceding 24 months, or women who have not undergone surgical sterilization, specifically hysterectomy, or bilateral oophorectomy and/or tubal ligation), will need a negative serum or urine pregnancy test within 48 hours prior to entry.

NOTE: Acceptable documentation of hysterectomy and bilateral oophorectomy, tubal ligation, tubal micro-inserts, vasectomy, and menopause is subject-reported history.

4.1.13 All subjects must agree not to participate in the conception process (e.g., active attempt to become pregnant or to impregnate, sperm donation, in vitro fertilization), and if participating in sexual activity that could lead to pregnancy, the subject/ partner must use at least two reliable methods of contraception, (condoms, with or without a spermicidal agent; a diaphragm or cervical cap with spermicide; an IUD; or hormonal based contraception), while receiving study treatment and for 6 weeks after stopping study treatment.

4.1.14 Men and women age ≥18 years.

4.1.15 Ability and willingness of subject to provide informed consent.

## 4.2 Exclusion Criteria

4.2.1 Previously documented virologic failure on an antiretroviral regimen. Previously documented virologic failure is defined as two confirmed HIV-1 RNA levels ≥400 copies/mL after achieving a viral load of <400 copies/mL during a time period in which the subject reports taking ART or failure to achieve a viral load <400 copies/mL after 6 months of ART.

4.2.2 Unstable clinical condition, such as unstable cardiac disease, or cancer requiring ongoing chemotherapy or radiation therapy, or other medical condition which, in the opinion of the investigator, would preclude a subject from safely undergoing study procedures.

4.2.3 Breast-feeding or pregnancy.

4.2.4 Use of immunosuppressive medications within 60 days prior to study entry, such as interferon-alpha, cancer chemotherapy, corticosteroid therapy equal to or exceeding a dose of 15 mg/day of prednisone for more than 10 days

NOTE: Use of inhaled or nasal steroid use is not exclusionary.

4.2.5 An opportunistic infection within 60 days prior to entry.

4.2.6 Known allergy/sensitivity or any hypersensitivity to components of study drug(s) or their formulation.

4.2.7 Active drug or alcohol use or dependence that, in the opinion of the site investigator, would interfere with adherence to study requirements.

4.2.8 Serious illness requiring systemic treatment and/or hospitalization within 60 days prior to entry.

4.2.9 Receipt of a non-HIV vaccination within 30 days prior to study entry.

4.2.10 Receipt of any HIV vaccines.

4.2.11 Plan to change the background ART within 24 weeks after study entry.

## 4.3 Study Enrollment Procedures

4.3.1 Prior to implementation of this protocol, sites must have the protocol and protocol consent forms approved by their local institutional review board (IRB). Protocol documents must be registered with and approved by the DAIDS Regulatory Compliance Center (RCC) Protocol Registration Office. Protocol registration must occur before the site can enroll any subjects into the study.

Once a candidate for study entry has been identified, details will be carefully discussed with the subject. The subject will be asked to read and sign the approved protocol consent form.

For subjects from whom a signed informed consent has been obtained, an ACTG Screening Checklist must be entered through the DMC Subject Enrollment System.

4.3.2 Prisoner Participation

DAIDS has concluded that this protocol does NOT meet Federal requirements governing prisoner participation in clinical trials and should NOT be considered by local IRBs for the recruitment of prisoners.

4.3.3 Randomization/Registration

At entry, subjects will be enrolled to the study according to standard ACTG Data Management Center procedures.

For subjects from whom informed consent has been obtained, but who are deemed ineligible or who do not enroll into the initial protocol step, an ACTG Screening Failure Results form must be completed and keyed into the database.

## 4.4 Coenrollment Guidelines

Sites are strongly encouraged to coenroll subjects in A5128. Coenrollment in A5128, “Plan for Obtaining Informed Consent to Use Stored Human Biological Materials (HBM) for Currently Unspecified Analyses,” does not require permission from the A5244 protocol chair. For specific questions and approval for coenrollment in other studies, sites must contact the protocol chair via e-mail as described in the Study Management section.

# 5.0 STUDY TREATMENT

Study treatment is defined as raltegravir (MK-0518) and raltegravir placebo.

## 5.1 Regimens, Administration, and Duration

At study entry the subjects will be randomized to one of the following arms:

- Arm A (immediate intensification): At week 0, continue entry regimen (at least 2 NRTIs + PI or NNRTI [not provided by the study]) and add raltegravir 400 mg PO BID for 12 weeks. At week 12, stop raltegravir and add placebo BID for 12 weeks.
- Arm B (delayed intensification): At week 0, continue entry regimen (at least 2 NRTIs + PI or NNRTI [not provided by the study]) and add placebo BID for 12 weeks. At week 12, stop placebo and add raltegravir 400 mg PO BID for 12 weeks.

At the entry visit, the pharmacist will dispense one bottle of study product. At the week 4 visit, any remaining tablets will be collected from the participant and the pharmacist will dispense two bottles of study product. At the week 12 visit the participant must return any remaining study product. At this visit, the pharmacist must receive a new prescription to ensure that the regimen switch occurs. The pharmacist will dispense one bottle at this visit. At the week 16 visit, any remaining tablets will be collected from the participant and the pharmacist will dispense two bottles of study product. At the week 24 visit, the participant must return any remaining study product.

## 5.2 Study Product Formulation and Preparation

Raltegravir (MK-0518) 400-mg tablets and placebo for raltegravir must be dispensed in the original bottle with the desiccant provided. Store at room temperature (25°C, 77°F) and protect from moisture.

## 5.3 Pharmacy: Product Acquisition, Distribution, and Accountability

5.3.1 Study Product Acquisition/Distribution

Raltegravir and placebo supplied by Merck and Company will be available through the NIAID Clinical Research Products Management Center (CRPMC). The site pharmacist can obtain the study products for this protocol by following the instructions in the manual *Pharmacy Guidelines and Instructions for DAIDS Clinical Trials Networks* in the section Study Product Control.

5.3.2 Study Product Accountability

The site pharmacist is required to maintain complete records of all study products received from the NIAID CRPMC and subsequently dispensed. All unused study products must be returned to the NIAID CRPMC (or as otherwise directed by the sponsor) after the study is completed or terminated. The procedures to be followed are provided in the manual *Pharmacy Guidelines and Instructions for DAIDS Clinical Trials Networks* in the section Study Product Control.

## 5.4 Concomitant Medications

To avoid AEs caused by drug interactions, sites must refer to the most recent package inserts for study drugs and concomitant agents whenever a concomitant medication is initiated or a dose is changed.

Sites must also refer to the study product’s most recent package insert or investigator brochure to access additional current information on prohibited and precautionary medications.

Below are lists of selected concomitant medications. These lists are only current as of the date of this protocol. Therefore, whenever a concomitant medication or study agent is initiated or a dose changed, investigators must review the concomitant medications’ and study agents' most recent package inserts, investigator's brochure, or updated information from the DAIDS to obtain the most current information on drug interactions, contraindications, and precautions.

The ACTG Pharmacology Committee updated drug table is located at <http://aactg.s-3.com/members/aphinfo.htm>.

5.4.1 Required Medications

Subjects must be on combination ART as specified in the inclusion criteria.

5.4.2 Prohibited Medications

The prohibited medications with raltegravir are dilantin, phenobarbitol and rifampin.

Receipt of a vaccination while on study is prohibited.

Table 3: Prohibited with NNRTI Medications

| Medication Class | Prohibited Medications |
| --- | --- |
| Alternative/Complementary | St. John’s wort (*Hypericum perforatum*) |
| Antihistamines | astemizole |
|  | terfenadine |
| Antiarrhythmics | bepridil^1^ |
| Antimigraine Agents | eletriptan^1^  dihydroergotamine  ergonovine  ergotamine  methylergonovine |
| GI Motility | cisapride |
| Sedative/hypnotics | midazolam^2^ |
|  | triazolam |
| Anti-infectives | ketoconazole  voriconazole |
| HMG CoA Reductase Inhibitors | lovastatin  simvastatin |
| Psychiatric Medications | pimozide^1^ |

^1^ Prohibited only with EFV.

^2^ A dose of midazolam is allowed for a procedure with appropriate monitoring of vital signs.

Table 4: Prohibited with PI Medications

| Medication Class | PI Agents |
| --- | --- |
| H2 Blockers, Proton Pump Inhibitors^1^ | H2 Blockers:  cimetidine  famotidine  nizatidine  ranitidine |
|  | Proton Pump Inhibitors:  esomeprazole  lansoprazole  omeprazole  pantoprazole  rabeprazole |
| Antiarrhythmics | amiodarone |
|  | flecainide |
|  | propafenone |
|  | quinidine  bepridil |
| Antihistamines | astemizole |
|  | terfenadine |
| Anti-infectives | systemic itraconazole |
|  | rifampin, rifapentine  voriconazole |
| Antimigraine Agents | eletriptan^2^ |
| GI Motility | cisapride |
| HMG-CoA Reductase Inhibitors | lovastatin |
|  | simvastatin |
| Psychiatric Medications | pimozide |
| Alternative/Complementary | St. John’s wort (*Hypericum perforatum*) |
| Sedative/hypnotics | midazolam^3^ |
|  | triazolam |
| Other | alfuzosin |
|  | dihydroergotamine |
|  | ergonovine |
|  | ergotamine |
|  | methylergonovine |

^1^Prohibited only with ATV. Antacids may be used but need to be dosed separate from ATV.

^2^ Prohibited only with RTV.

^3^A dose of midazolam is allowed for a procedure with appropriate monitoring of vital signs.

5.4.3 Precautionary Medications

See the protocol-specific web site (<http://aactg.s-3.com/members/ps/5244/ps5244.htm>) for a complete list of precautionary medications. Package inserts of antiretroviral drugs and concomitant agents should be referenced whenever a concomitant medication is initiated or dose changed, to avoid drug interaction AEs.

## 5.5 Adherence Assessment

Adherence will be assessed by pill count for raltegravir/placebo at every visit starting at week 2. In addition, the standard ACTG adherence questionnaire will be administered at weeks 4, 12, 16, and 24, as indicated in the schedule of events.

6.0 CLINICAL AND LABORATORY EVALUATIONS

## 6.1 Schedule of Events

|  |  |  |  | Post-Entry Evaluations  (Weeks) | | | | | | | | Confir-mation of virologic failure | Premature Study Disconti-nuation Evaluations |
| --- | --- | --- | --- | --- | --- | --- | --- | --- | --- | --- | --- | --- | --- |
| Evaluation | Screening | Pre-Entry | Entry | 2 | 4 | 10 | 12 | 14 | 16 | 22 | 24 |
| Documentation of HIV | X |  |  |  |  |  |  |  |  |  |  |  |  |
| Medical History/Medication History | X | X | X |  |  |  |  |  |  |  |  |  |  |
| Complete Physical Exam | X |  |  |  |  |  |  |  |  |  |  |  |  |
| Targeted Physical Exam |  | X | X | X | X | X | X | X | X | X | X |  | X |
| Hematology | X | X | X |  | X |  | X |  | X |  | X |  | X |
| Chemistry & Liver Function Tests | X |  | X |  | X |  | X |  | X |  | X |  | X |
| Pregnancy Test | X | If preg-nancy sus-pected | X | If pregnancy suspected | | | X | If pregnancy suspected | | | |  |  |
| CD4+/CD8+ | X | X | X |  |  |  | X |  |  |  | X |  | X |
| Advanced Flow (activated CD4+/ CD8+ cells) |  | X | X |  |  |  | X |  |  |  | X |  | X |
| HIV-1 RNA (real time) | X | X | X |  | X |  | X |  | X |  | X | X | X |
| HIV-1 RNA by SCA (real time) | X |  |  |  |  |  |  |  |  |  |  |  |  |
| HIV-1 RNA by SCA (stored plasma) |  | X | X | X | X | X | X | X | X | X | X |  | X |
| Historical Pre-ART Stored Plasma (if available; not required for entry into study; see section 6.2.1) |  | X |  |  |  |  |  |  |  |  |  |  |  |
| Stored Plasma and Cryopreserved PBMC |  | X | X |  |  |  | X |  |  |  | X |  | X |
| Stored Sample for Trough Raltegravir Level (see section 6.3.10 and Appendix I) |  |  |  |  |  | X | X |  |  | X | X |  |  |
| Dispense Study Drug |  |  | X |  | X |  | X |  | X |  |  |  |  |
| Pill Count |  |  |  | X | X | X | X | X | X | X | X |  | X |
| Adherence Questionnaire |  |  |  |  | X |  | X |  | X |  | X |  |  |

## 6.2 Timing of Evaluations

6.2.1 Screening and Pre-Entry Evaluations

Screening and pre-entry evaluations must occur prior to the subject’s starting any study medications.

Screening

Screening evaluations to determine eligibility must be completed within 60 days prior to study entry unless otherwise specified.

In addition to data being collected on subjects who enroll into the study, demographic, clinical, and laboratory data on screening failures will be captured in a screening log and entered into the ACTG database.

Pre-Entry

Pre-entry evaluations must be completed within 14 days prior to study entry, unless otherwise specified. Screening and pre-entry visits must be separated by at least 24 hours.

Confirmation of the availability of the stored pre-entry plasma sample for HIV-1 RNA SCA determination is required in order to enroll subjects into the study. The site must confirm that this sample has been entered into the LDMS.

If a subject has a frozen plasma sample available prior to initiation of ART, the sample should be shipped as outlined in Appendix I. If this is an ACTG sample stored at BRI, the team will request the sample through appropriate mechanisms. If the sample is stored locally, then the site must ship it to BRI. The purpose of this sample is to allow for the assessment of amplification efficiency of the SCA on stored samples. This sample is not required for entry into the study.

6.2.2 Entry Evaluations

Entry evaluations must occur at least 24 hours after pre-entry evaluations. Subject must begin treatment within 72 hours after registration/randomization.

6.2.3 Post-Entry Evaluations

Confirmation of the availability of the stored week 10 plasma sample for HIV-1 RNA SCA determination is required prior to starting raltegravir/placebo at week 12. The site must confirm that this sample has been entered into the LDMS.

Visit to Confirm a Suspected Virologic Failure

Virologic failure is defined as two consecutive HIV-1 RNA levels ≥50 copies/mL by real-time HIV-1 RNA testing. Subjects with a plasma HIV-1 RNA ≥50 copies/mL at any visit will have a confirmatory viral load obtained as soon as possible and within 4 weeks after the first sample was drawn, if possible. If this visit coincides with a regularly scheduled visit, the evaluations should be combined.

Subjects confirmed to have virologic failure will no longer contribute data to the primary outcome. Upon confirmation of virologic failure, raltegravir will be discontinued and the subject will complete the week 12 and 24 study visit evaluations, except for the collection of samples for HIV-1 RNA by SCA test which will not be performed, and then be taken off study.

- - 1. Discontinuation Evaluations

Evaluations for Randomized or Registered Subjects Who Do Not Start Study Treatment

Subjects who do not start study treatment will be taken off study with no further evaluations required. The subject will be replaced. All case report forms (CRFs) must be completed and keyed for the period up to and including week 0.

Premature Study Treatment/Background ART Discontinuation Evaluations

Subjects who discontinue raltegravir/placebo due to an AE will be followed. The subject will complete the week 12 and 24 evaluations, as listed in the Schedule of Events (section 6.1), except for the collection of samples for HIV-1 RNA by SCA, which will not be performed and then be taken off study.

Subjects who discontinue raltegravir/placebo for more than 14 days or who have confirmed virologic failure will complete the week 12 and 24 evaluations, as listed in the Schedule of Events (section 6.1), except for the collection of samples for HIV-1 RNA by SCA, which will not be performed and then be taken off study.

Subjects who discontinue their background ART entry regimen for ≥14 consecutive days must discontinue study treatment (Raltegravir) and will complete the week 12 and 24 evaluations, as listed in the Schedule of Events, except for the collection of samples for HIV-1 RNA by SCA, which will not be performed and then be taken off study.

Premature Study Discontinuation Evaluations

Subjects who prematurely withdraw from the study will have the premature discontinuation evaluations performed as listed in the Schedule of Events and then be taken off study.

Study Completion Evaluations

The week 24 evaluations will be completed as the subject’s final on-study visit.

6.2.5 Pregnancy

Subjects who become pregnant after study entry must discontinue study treatment immediately. Subjects will continue to be followed on study/off study treatment as per the Schedule of Events in Section 6.1, except that the following will not be performed:administration of study medication, pill count and adherence questionnaires, HIV-1 RNA by SCA, advanced flow, stored samples for trough raltegravir level, and plasma and cryopreserved PBMC. The core team must be notified of any pregnancies that occur in subjects on study ([actg.corea5244@fstrf.org](mailto:actg.corea5244@fstrf.org)).

Pregnancies that occur on study should be reported to The Antiretroviral Pregnancy Registry. More information is available at [www.apregistry.com](http://www.apregistry.com/). Phone: 800-259-4263; Fax: 800-800-1052.

Intrapartum complications and/or pregnancy outcome will be recorded on the CRFs up to week 24 and also be reported to The Antiretroviral Pregnancy Registry. After week 24, intrapartum complications and/or pregnancy outcome will be reported only to The Antiretroviral Pregnancy Registry.

## 6.3 Instructions for Evaluations

All clinical and laboratory information required by this protocol is to be present in the source documents. Sites must refer to the Source Document Guidelines on the ACTG Web site for information about what must be included in the source document: <http://rcc.tech-res-intl.com/members/download/SourceDocSOP.pdf>

All stated evaluations are to be recorded on the CRF and keyed into the database unless otherwise specified.

6.3.1 Documentation of HIV-1

HIV-1 infection, as documented by any licensed ELISA test kit and confirmed by Western blot at any time prior to study entry. HIV-1 culture, HIV-1 antigen, plasma HIV-1 RNA, or a second antibody test by a method other than ELISA is acceptable as an alternative confirmatory test.

6.3.2 Medical History

The medical history must include all diagnoses identified by the ACTG criteria for clinical events and other diagnoses. Any allergies to any medications and their formulations must be documented. For current criteria, refer to the appendix identified in the study CRF. [http://www.fstrf.org/ACTG/appendices/appendices.html](http://www.fstrf.org/ACTG/appendices/appendicies.html)).

6.3.3 Medication History

A medication history must be present, including start and stop dates (estimated if the exact dates cannot be obtained). The table below lists the medications that must be included in the history.

Table 5: Medication History Table

| Medication Category | Complete History or Timeframe |
| --- | --- |
| Antiretroviral therapy | Complete History |
| Immune-based therapy | Within 60 days prior to entry |
| Blinded study treatment | Complete History |
| HIV-1-related vaccines | Complete History |
| Prescription drugs for treatment of opportunistic infections | Within 60 days prior to entry |
| Prescription drugs for prophylaxis of opportunistic infections | Within 60 days prior to entry |
| Prescription and non-prescription drugs | Within 60 days prior to entry |

6.3.4 HIV-1 RNA History

Record the documented date and viral load of the subject’s first HIV-1 RNA level that was undetectable. If documentation is not available, then subject recollection will be allowed.

6.3.5 Clinical Assessments

Complete Physical Exam

A complete physical examination is required at screening and includes an examination of the skin, head, mouth, and neck; auscultation of the chest; cardiac exam; abdominal exam; and examination of the lower extremities for edema*.* The complete physical exam will also include signs and symptoms, diagnoses, vital signs (temperature, pulse, respiration rate*,* and blood pressure), height, and weight.

Targeted Physical Exam

A targeted physical examination at pre-entry, entry, and all post entry visits is to be driven by any previously identified or new signs or symptoms that the subject has experienced since the last visit. This exam also includes weight, vital signs (temperature, pulse, respiration rate, and blood pressure), and diagnoses at all visits.

Height

Height will be recorded on the CRF at the screening visit only.

Signs and Symptoms

At entry, record all signs and symptoms occurring 30 days prior to entry. After entry, record signs and symptoms ≥ Grade 2. Any signs or symptoms that led to a change in treatment (raltegravir/placebo), regardless of grade, must be recorded.

Sites must refer to the Division of AIDS Table for Grading the Severity of Adult and Pediatric Adverse Events (DAIDS AE Grading Table), Version 1.0, December 2004, which can be found on the DAIDS RCC Web site: [http://rcc.tech-res-intl.com/eae/htm](http://rcc.tech-res-intl.com/)

Diagnoses

Record all diagnoses identified by the ACTG criteria for clinical events and other diseases. All confirmed and probable diagnoses made since the last visit will be recorded.

Concomitant Medications

All concomitant medications taken since the last visit will be recorded in the source documents.

Please note that only prescription medications will be recorded on the CRFs.

Antiretroviral Medications

All modifications to antiretroviral medications including initial doses, subject-initiated and/or protocol-mandated interruptions, modifications, and permanent discontinuation will be recorded on the CRFs.

Study Treatment Modifications

All modifications to study treatment, including initial dose, subject-initiated and/or protocol-mandated modifications, and permanent discontinuation will be recorded on the CRFs. If a subject misses more than 4 consecutive doses, then study treatment is considered modified and this must be reported appropriately on the CRF.

Intrapartum Complications and/or Pregnancy Outcome

Record all intrapartum complications and/or pregnancy outcome occurring since the last visit on the CRFs.

6.3.6 Laboratory Evaluations

At screening, pre-entry, and entry, all laboratory values, regardless of grade, must be recorded on the CRFs. For post-entry assessments, record all laboratory values Grade 3. Any laboratory value that led to a change in treatment (raltegravir/placebo), regardless of grade, must be recorded.

Sites must refer to the Division of AIDS Table for Grading the Severity of Adult and Pediatric Adverse Events (DAIDS AE Grading Table), Version 1.0, December 2004, which can be found on the DAIDS RCC Web site: [http://rcc.tech-res-intl.com/eae/htm/](http://rcc.tech-res-intl.com/)

Hematology

Hemoglobin, hematocrit, red blood cells (RBC), white blood count (WBC), differential WBC, absolute neutrophil count (ANC), platelets.

Blood Chemistries:

Sodium, potassium, bicarbonate, chloride, blood urea nitrogen, creatinine, glucose.

Liver Function Tests

ALT [SGPT], AST [SGOT], total bilirubin, direct bilirubin, and indirect bilirubin, alkaline phosphatase.

Pregnancy Test

For women with reproductive potential: Serum or urine β-HCG (urine test must have a sensitivity of 25-50 mIU/mL). A pregnancy test will be done at screening, entry, week 12 and whenever pregnancy is suspected. If the pregnancy test is positive at entry, the subject will come off the study and will be replaced.

6.3.7 Immunologic Studies

CD4+/CD8+

Obtain absolute CD4+ and CD8+ count and percentages within 60 days prior to entry from a laboratory that possesses a CLIA certification or equivalent.

During the study, all laboratories must possess a CLIA certification or equivalent and must be certified for protocol testing by the DAIDS Immunology Quality Assurance (IQA) Program.

Evaluations for CD4+ and CD8+ counts and subset percentage evaluations should be performed at the same ACTG-certified laboratory throughout the course of the study.

Because of the diurnal variation in CD4+ and CD8+ cell counts, determinations for individual subjects should be obtained consistently in either the morning or the afternoon throughout the study, if possible.

NOTE: Each time a CD4+ and CD8+ measurement is obtained, the local laboratory must perform a WBC and differential from a sample obtained at the same time.

Advanced Flow (Activated CD4+ and CD8+ cells)

Activated CD4+: Percentage of CD4+, HLA-DR+, CD38+ T-cells.

Activated CD8+: Percentage of CD8+, HLA-DR+, CD38+ T-cells.

6.3.8 Virologic Studies

Plasma HIV-1 RNA (real time)

Screening HIV-1 RNA must be performed within 60 days prior to study entry by a laboratory that possesses a CLIA certification or equivalent. Eligibility will be determined based on the screening value.

HIV-1 RNA quantitation will be done in real time. All HIV-1 RNA specimens will be sent to the protocol designated virology laboratory (see Appendix I).

Plasma HIV-1 RNA by SCA (real time and stored)

This test will be performed at screening, pre-entry, entry, and as required in the Schedule of Events. Only the screening SCA test must be performed real time within 60 days prior to entry.

Please see Appendix I for details on processing plasma samples for the SCA test.

Historical Pre-ART Stored Plasma (if available; this sample is not required for entry into study; see section 6.2.1)

The pre-ART frozen plasma sample should be shipped as outlined in Appendix I. If this is an ACTG sample stored at BRI, the team will request the sample through appropriate mechanisms. If the sample is stored locally, then the site must ship it to BRI.

6.3.9 Stored Plasma and Cryopreserved PBMC

Samples will be stored at BRI for additional immunology and virology testing. See Appendix I for details.

6.3.10 Pharmacokinetic Studies – Raltegravir Trough Level

Blood will be drawn at the visits designated in the Schedule of Events prior to dosing of raltegravir and stored for future exploratory analysis. The last dose of raltegravir should be taken 8-12 hours before the trough level is obtained. See Appendix I for details of sample processing.

6.3.11 Pill Count for Raltegravir/Placebo

The number of pills initially dispensed will be recorded on the CRFs and the pill count will be performed as indicated in Section 6.1.

6.3.12 Adherence Questionnaires

Subjects will need approximately 10 additional minutes to complete the brief ACTG adherence questionnaire. The questionnaire is not required of subjects who discontinue the study drug.

# 7.0 CLINICAL MANAGEMENT ISSUES

Criteria for subject management, dose interruptions, modifications, and discontinuation changes in drug treatment will be mandated only for toxicities attributable to raltegravir. Toxicities due to drugs in the background regimen should be managed according to standard clinical practice, with the goal of maintaining continuous therapy, if possible.

The grading system for drug toxicities is located in the Division of AIDS Table for Grading the Severity of Adult and Pediatric Adverse Events, Version 1.0, December 2004, located at the DAIDS RCC web site: <http://rcc.tech-res-intl.com/eae.htm>.

NOTE: The core team must be notified by e-mail at [actg.corea5244@fstrf.org](mailto:actg.corea5244@fstrf.org) regarding toxicities that result in a change in regimen.

## 7.1 Toxicity Management

7.1.1 Grade 1 or 2 Toxicity

Subjects who develop a Grade 1 or 2 AE or toxicity may continue raltegravir. If a subject chooses to discontinue raltegravir, the site should complete regimen change evaluations, notify the A5244 core team ([actg.corea5244@fstrf.org](mailto:actg.corea5244@fstrf.org)), and encourage the subject to complete safety visits for the study until the toxicity resolves.

7.1.2 Grade 3 Toxicity

If the investigator has compelling evidence that the AE has NOT been caused by raltegravir, dosing may continue. Subjects who develop a Grade 3 AE or toxicity, except as stated in the following sections, should have their raltegravir withheld and the A5244 core team must be consulted. The subject should be reevaluated closely until the AE returns to Grade ≤2, at which time raltegravir may be reintroduced at the discretion of the site investigator or according to standard practice.

If the same Grade 3 AE recurs within 4 weeks, the raltegravir must be permanently discontinued. If the same Grade 3 AE recurs after 4 weeks but is not believed to be related to raltegravir, the management scheme outlined above may be repeated. If the same Grade 3 AE recurs and is thought to be possibly due to raltegravir, the raltegravir must be permanently discontinued.

Subjects experiencing Grade 3 AEs requiring permanent discontinuation of raltegravir should be followed closely for resolution of the AE to Grade ≤2 and the core team must be consulted.

Subjects with Grade 3 asymptomatic laboratory abnormalities in cholesterol, creatine kinase (CK), or triglycerides may continue raltegravir.

7.1.3 Grade 4 Toxicity

Subjects who develop a Grade 4 symptomatic AE or toxicity will have raltegravir discontinued. If the site investigator has compelling evidence that the AE has not been caused by raltegravir, dosing may resume when the AE has resolved and after consulting with the core team. Subjects experiencing Grade 4 AEs requiring permanent discontinuation of raltegravir should be followed closely until resolution of the AE to Grade ≤2 and the core team must be consulted.

Subjects with Grade 4 asymptomatic laboratory abnormalities in cholesterol, CK, or triglycerides may continue raltegravir.

7.1.4 Rash

7.1.4.1 Grade 2

Antihistamines, topical corticosteroids, or a brief course of systemic corticosteroids, at the discretion of the site investigator, may be prescribed and subject may continue raltegravir. The subject should be advised to contact the physician immediately if there is any worsening of the rash, or if systemic signs or symptoms develop that could be compatible with a hypersensitivity reaction (HSR).

If the rash is considered to be most likely due to concomitant illness or drug, standard management, including discontinuation of the likely causative agent, should be undertaken. If no other causative factor is found after clinical evaluation, the subject should be treated symptomatically until the rash resolves.

7.1.4.2 Grade 3

If the rash is thought to be related to raltegravir, subjects must discontinue study drug.

7.1.4.3 Grade 4

Subjects must discontinue raltegravir. The core team must be consulted.

In the event that Grade 2, 3, or 4 rash fails to resolve, increases in severity, is associated with systemic (e.g., fever, malaise, nausea) or allergic (e.g., urticaria) symptoms or Grade 3 or 4 LFT elevations, or is associated with exfoliative dermatitis or mucous membrane involvement or erythema multiforme or suspected Stevens-Johnson syndrome or necrosis requiring surgery, raltegravir should be discontinued until symptom resolution. The core team must be consulted.

7.1.5 Clinical Pancreatitis

Clinical pancreatitis is not commonly associated with the study-provided drug. If clinical pancreatitis is suspected, please refer to the ACTG Definition of Pancreatitis, located at <http://aactg.s-3.com/members/download/other/pancdefv2.doc>. The core team must be consulted.

7.1.6 Nausea/Vomiting

Nausea/vomiting may be treated symptomatically with oral antiemetics or antiemetic suppositories. For Grade ≥3 nausea and vomiting thought secondary to raltegravir that fails to improve on antiemetics to Grade ≤2, raltegravir may be held until Grade ≤ 2. If Grade ≥3 nausea and vomiting recurs with reinstitution of raltegravir or persists beyond 14 days despite symptomatic management, then raltegravir may be discontinued at the discretion of the investigator in consultation with the core team.

7.1.7 Diarrhea

Symptomatic treatment of any grade diarrhea with oral antidiarrheal drugs is permitted once infectious causes of diarrhea have been ruled out by the site investigator. If Grade ≥3 diarrhea thought to be related to raltegravir persists despite symptomatic management, then raltegravir should be held until Grade ≤2. If Grade ≥3 diarrhea recurs with reinstitution of raltegravir or persists beyond 14 days despite symptomatic management, then raltegravir should be discontinued.

7.1.8 Hypertriglyceridemia/Hypercholesterolemia

Subjects who experience asymptomatic triglyceride or cholesterol elevations may continue to receive raltegravir. Only triglyceride levels done in a fasting state should be graded for toxicity. For Grade ≥3, confirmatory fasting triglyceride should be obtained within 4 weeks and must be obtained prior to the institution of medical therapy for the hyperlipidemia.

Subjects with asymptomatic triglyceride or cholesterol elevation should be counseled on dietary modification. Oral antihyperlipidemic agents should be considered at the discretion of the site investigator (and documented on the concomitant drug CRF). Guidelines for the treatment of hyperlipidemia are available on the ACTG Web site (<http://aactg.s-3.com/members/download/other/metabolic/Chol.rtf>). Resin binding agents, such as cholestyramine and colestipol, should be avoided as they may interfere with absorption of PIs as well as the absorption of other drugs and fat-soluble vitamins.

7.1.9 Hyperglycemia

Fasting hyperglycemia >110 to 125 mg/dL is considered evidence of impaired glucose tolerance. A fasting blood glucose level above 126 mg/dL is highly suggestive of diabetes mellitus. Subjects with fasting hyperglycemia (of any grade) may continue raltegravir at the discretion of the investigator in consultation with the core team. A confirmatory fasting blood glucose level for newly diagnosed hyperglycemia must be obtained within 4 weeks and prior to the institution of medical therapy. Hyperglycemia may be treated with oral hypoglycemic agents or insulin according to standard guidelines.

7.1.10 AST/ALT Elevations

7.1.10.1 Grade 3

Raltegravir may be continued for asymptomatic, isolated Grade 3 AST or ALT elevations at the discretion of the site investigator. Careful assessments should be done to rule out the use of alcohol, non- raltegravir -related drug toxicity, or viral hepatitis as the cause of the Grade 3 elevation.

For symptomatic elevations of AST or ALT, raltegravir should be stopped and the core team must be consulted.

7.1.10.2 Grade 4

Raltegravir should be held for AST or ALT Grade 4 elevations until the toxicity returns to Grade ≤2. If the Grade 4 elevation in AST or ALT recurs on rechallenge, raltegravir should be permanently discontinued.

Any symptomatic Grade 3/4 ALT or AST elevation should lead to permanent discontinuation if it is suspected to be due to raltegravir.

# 8.0 CRITERIA FOR DISCONTINUATION

## 8.1 Permanent Study Drug Discontinuation

- Drug-related toxicity requiring permanent discontinuation (see Section 7.1).
- Requirement for prohibited concomitant drugs (see Section 5.4).
- Request by subject to terminate treatment.
- Confirmation of virologic failure (see Section 6.2.3).
- Clinical reasons believed life threatening by the physician, even if not addressed in the toxicity section of the protocol.
- Subject repeatedly noncompliant with raltegravir as prescribed.
- Subject discontinues raltegravir/placebo for ≥14 consecutive days.
- Subject discontinues background entry regimen for ≥14 consecutive days.
- Failure by the subject to attend ≥3 consecutive clinic visits.
- Pregnancy or breast-feeding.

## 8.2 Premature Study Discontinuation

- Request by the subject to withdraw.
- Request of the primary care provider if s/he thinks the study is no longer in the best interest of the subject.
- Subject judged by the investigator to be at significant risk of causing harm to self.
- At the discretion of the ACTG, IRB, Food and Drug Administration (FDA), Office for Human Research Protections (OHRP), NIAID, investigator, or pharmaceutical supporter.

# 9.0 STATISTICAL CONSIDERATIONS

## 9.1 General Design Issues

This is a randomized, double-blinded, placebo-controlled cross-over pilot study to measure the effect of treatment intensification with raltegravir on the level of plasma HIV-1 RNA level in subjects who have a HIV-1 RNA level <50 copies/mL on PI- or NNRTI-containing regimens.

At study entry, the subjects will be randomized with equal likelihood to the two arms, using permuted blocks. Subjects randomized to Arm A will add raltegravir to their ART regimen for 12 weeks and at week 12 stop raltegravir and add placebo for 12 weeks. Subjects randomized to Arm B will add placebo first to their ART regimen for 12 weeks and at week 12 stop placebo and add raltegravir for 12 weeks.

## 9.2 Endpoints

9.2.1 Primary Endpoint

The average of log_10_ HIV-1 RNA, as measured by real-time PCR single copy SCA, at weeks 10 and 12 (or the last 2 scheduled evaluations after week 8 and before switching regimen to either placebo [Arm A] or raltegravir [Arm B]). If these 2 evaluations are more than 3 weeks apart, instead of calculating an average, the evaluation closest to week 12 will be used.

9.2.2 Secondary Endpoints

9.2.2.1 Log_10_ HIV-1 RNA level as measured by SCA at baseline (defined as the average of pre-entry and entry values), at weeks 10/12 (defined as the average of weeks 10 and 12 values), and weeks 22/24 (defined as the average of weeks 22 and 24 values).

9.2.2.2 CD4+ T-cell count at baseline (defined as the average of pre-entry and entry values), and at weeks 12 and 24.

9.2.2.3 CD8+ T-cell count at baseline (defined as the average of pre-entry and entry values), and at weeks 12 and 24.

9.2.2.4 The level of CD4+ T-cell activation, defined as percent CD4+ HLA-DR+, CD38+, at baseline (defined as the average of pre-entry and entry values), and at weeks 12 and 24.

9.2.2.5 The level of CD8+ T-cell activation, defined as percent CD8+ HLA-DR+, CD38+ at baseline (defined as the average of pre-entry and entry values), and at weeks 12 and 24.

9.2.2.6 Occurrence of a study related AE (defined as Grade ≥2 signs/symptoms, Grade ≥3 laboratory abnormalities, and clinical events) that are “possibly”, “probably” or “definitely” related to study treatment any time from the first day of study treatment to week 12 and from week 12 to week 24.

9.2.2.7 The proportion of subjects who discontinue study drug prior to week 12 and to week 24.

## 9.3 Randomization and Stratification

Subjects will be randomized with equal probability to the two arms of the study. The randomization will use a stratified permuted blocks approach. Stratification will be by current ART regimen (PI- or NNRTI-containing).

## 9.4 Sample Size and Accrual

Twenty-five subjects will be enrolled in each treatment group (total enrollment: 50 subjects). It is estimated that four subjects per arm will not contribute to the primary outcome (e.g., because of discontinuing ART, having virologic failure, or discontinuing study drug). With 18 evaluable subjects in each treatment group with weeks 10/12 SCA results, there would be 80% power to detect a 0.5 log_10_ effect of intensification (1 standard deviation effect size) using Wilcoxon rank-sum test. The number of evaluable subjects has been inflated by 15% (from 18 to 21 subjects) to account for the fact that intensification may increase the proportion of subjects who are below the limit of the SCA at weeks 10/12, which impacts statistical power^(12)^. In calculating power, the standard deviation of SCA is estimated based on information provided by M. King^(13)^.

It is anticipated that it will take 6-12 months to enroll the study (50 subjects). Each subject will be followed for 24 weeks.

## 9.5 Monitoring

Accrual and a summary of all Grade ≥2 signs and symptoms and all Grade ≥3 laboratory abnormalities will be reviewed by the team monthly. The AE summary will be pooled over the treatment arms. In addition, baseline characteristics as well as early study treatment and study discontinuations, pooled over treatment arms, will be reviewed regularly by the team. SDAC will also prepare a quarterly report of all AEs by blinded treatment arm, to be reviewed quarterly by the DAIDS Clinical representative or designee. Approximately one year after enrollment of the first subject, an interim review of the study will occur. A Translational Research and Drug Development (TRADD) SMC will review accrual, AE summaries, and off-treatment and off-study rates broken down by randomized treatment arms. A SMC may also be convened if a reason is identified by the DAIDS Clinical Representative, study chair, or study statistician in consultation with the team.

Unblinding of study treatment will be permitted only in instances of an emergency in which this information is essential to ensure appropriate care for the subject, as determined by the protocol Chair, Vice Chair, and the DAIDS clinical representative.  Unblinding procedures will follow the ACTG Standard Operation Procedure for Unblinding Subjects: <http://aactg.s-3.com/members/download/Final/asop/unblinding-subjects-sop-07.doc>.

## 9.6 Analyses

All statistical tests will be two-sided with nominal level of 0.05. Analysis will be exploratory without adjusting for multiple testing.

9.6.1 Primary Analysis

Comparison between treatment arms of the primary endpoint will be undertaken using the Wilcoxon rank-sum test. As a supplement, standard linear regression models that adjust for the stratification factor used in the randomization and for baseline HIV-1 RNA or CD4+ cell count, respectively, will be used for the comparison between treatment arms for the primary endpoint. If some subjects have HIV-1 RNA measurements below the limit of quantification of the assay, then regression models for censored data will be used. The primary analysis will be as-treated, excluding subjects who do not remain on the intensification regimen or subjects who do not have an observed primary endpoint. Subjects who discontinue raltegravir/placebo or background entry regimen for ≥ 14 consecutive days will no longer contribute data to the primary outcome after the date on which they first discontinued medication. In addition, subjects who have virologic failures will not be included in the analyses. A sensitivity analysis of evaluating changes in HIV-1 RNA will also be conducted by carrying forward the last observation when the subject is on study treatment. An intent-to-treat analysis will also be performed as a supplement.

9.6.2 Secondary analyses

9.6.2.1 HIV-1 RNA SCA

Change from baseline (defined as the average of pre-entry and entry values) to weeks 10/12 will be compared between arms using the Wilcoxon rank-sum test.

The change in HIV-1 RNA from pre-intensification level (Arm A: defined as baseline value; Arm B: defined as weeks 10/12 value) to post-intensification level (Arm A: defined as weeks 10/12 value; Arm B: defined as weeks 22/24 value) will be summarized by median change and 95% confidence interval on the median change.

The within-subject effect of intensification will be evaluated by contrasting the slope of HIV-1 RNA by SCA before versus after week 12.

NOTE: Subjects who discontinue the study drug or background entry regimen or experience virologic failure will not have SCA samples drawn (see section 6.2.4).

9.6.2.2 CD4+ and CD8+ cell count

CD4+ and CD8+ cell counts at week 12 and changes from baseline to week 12 will be compared between the arms by the Wilcoxon rank-sum test. The changes from pre-intensification level to post-intensification level will also be summarized by median change and 95% confidence interval on the median change.

9.6.2.3 CD4+ and CD8+ activation percent

Levels of CD4+ and CD8+ T-cell activation percent, defined as CD4+ and CD8+, CD38+/HLA-DR+, at week 12 and changes from baseline to week 12 will be compared between the arms by the Wilcoxon rank-sum test. The changes from pre-intensification level to post-intensification level will also be summarized by median change and 95% confidence interval on the median change.

9.6.2.4 Occurrence of study-related AE

The number of subjects who experience treatment-related AE from the first day of study treatment to week 12 and from week 12 to week 24 will be reported by arm.

9.6.2.5 Proportion of subjects who discontinue the study treatment

Proportion of subjects who discontinue the study treatment prior to week 12 and to week 24 will be summarized by arm. Reasons for discontinuing study drug will be listed.

# 10.0 PHARMACOLOGY PLAN

Not applicable

# 11.0 DATA COLLECTION AND MONITORING AND ADVERSE EVENT REPORTING

## 11.1 Records to Be Kept

CRFs will be provided for each subject. Subjects must not be identified by name on any CRFs. Subjects will be identified by the patient identification number (PID) and study identification number (SID) provided by the ACTG DMC upon randomization.

## 11.2 Role of Data Management

11.2.1 Instructions concerning the recording of study data on CRFs will be provided by the ACTG DMC. Each CTU and CRS is responsible for keying the data in a timely fashion.

11.2.2 It is the responsibility of the ACTG DMC to assure the quality of computerized data for each ACTG study. This role extends from protocol development to generation of the final study databases.

## 11.3 Clinical Site Monitoring and Record Availability

11.3.1 Site monitors under contract to the NIAID will visit participating CRSs to review the individual subject records, including consent forms, CRFs, supporting data, laboratory specimen records, and medical records (physicians’ progress notes, nurses’ notes, individuals’ hospital charts), to ensure protection of study subjects, compliance with the protocol, and accuracy and completeness of records. The monitors also will inspect sites’ regulatory files to ensure that regulatory requirements are being followed and sites’ pharmacies to review product storage and management.

- - 1. The site investigator will make study documents (e.g., consent forms, drug distribution forms, CRFs) and pertinent hospital or clinic records readily available for inspection by the local IRB, the site monitors, the FDA, the NIAID, the OHRP, and the pharmaceutical supporter(s) or designee for confirmation of the study data.

## 11.4 Expedited Adverse Event Reporting to DAIDS

The expedited adverse event (EAE) reporting requirements and definitions for this study and the methods for expedited reporting of AEs to the DAIDS RCC Safety Office are defined in the “Manual for Expedited Reporting of Adverse Events to DAIDS” (DAIDS EAE Manual), dated May 6, 2004. The DAIDS EAE Manual is available on the RCC Web site: [http://rcc.tech-res-intl.com/eae](http://rcc.tech-res-intl.com/).

AEs reported on an expedited basis must be documented on the DAIDS Expedited Adverse Event Reporting form (EAE Reporting Form) available on the RCC Web site: [http://rcc.tech-res-intl.com/eae](http://rcc.tech-res-intl.com/).

This study uses the standard level of expedited AE reporting as defined in the DAIDS EAE Manual.

The study agents that must be considered in determining relationships of AEs requiring expedited reporting to DAIDS are raltegravir or placebo.

The Division of AIDS Table for Grading the Severity of Adult and Pediatric Adverse Events (DAIDS AE Grading Table), Version 1.0, December 2004, must be used and is available on the DAIDS RCC Web site: [http://rcc.tech-res-intl.com/eae/htm](http://rcc.tech-res-intl.com/)/

AEs must be reported on an expedited basis at the standard level during the protocol-defined EAE Reporting Period, which is the entire study duration for an individual subject (from study enrollment until study completion or discontinuation of the subject from study participation for any reason).

After the end of the protocol-defined EAE Reporting Period stated above, sites must report serious, unexpected, clinical suspected adverse drug reactions if the study site staff becomes aware of the event on a passive basis, i.e., from publicly available information.

# 12.0 HUMAN SUBJECTS

## 12.1 Institutional Review Board (IRB) Review and Informed Consent

This protocol and the informed consent document (Appendix II) and any subsequent modifications will be reviewed and approved by the IRB or ethics committee responsible for oversight of the study. A signed consent form will be obtained from the subject. The consent form will describe the purpose of the study, the procedures to be followed, and the risks and benefits of participation. A copy of the consent form will be given to the subject and this fact will be documented in the subject’s record.

## 12.2 Subject Confidentiality

All laboratory specimens, evaluation forms, reports, and other records that leave the site will be identified by coded number only to maintain subject confidentiality. All records will be kept locked. All computer entry and networking programs will be done with coded numbers only. Clinical information will not be released without written permission of the subject, except as necessary for monitoring by IRB, the FDA, the NIAID, the OHRP, or the pharmaceutical supporter or designee.

## 12.3 Study Discontinuation

The study may be discontinued at any time by the ACTG, IRB, the NIAID, the pharmaceutical supporter, the FDA, the OHRP, or other government agencies as part of their duties to ensure that research subjects are protected.

# 13.0 PUBLICATION OF RESEARCH FINDINGS

Publication of the results of this trial will be governed by ACTG policies. Any presentation, abstract, or manuscript will be made available for review by the pharmaceutical supporter prior to submission.

# 14.0 BIOHAZARD CONTAINMENT

As the transmission of HIV and other blood-borne pathogens can occur through contact with contaminated needles, blood, and blood products, appropriate blood and secretion precautions will be employed by all personnel in the drawing of blood and shipping and handling of all specimens for this study, as currently recommended by the Centers for Disease Control and Prevention and the National Institutes of Health.

All dangerous goods materials, including diagnostic specimens and infectious substances, must be transported according to the instructions detailed in the International Air Transport Association (IATA) Dangerous Goods Regulations.

.

15.0 REFERENCES

(1) Finzi D, Blankson J, Siliciano JD et al. Latent infection of CD4+ T cells provides a mechanism for lifelong persistence of HIV-1, even in patients on effective combination therapy. Nat Med 1999; 5(5):512-517.

(2) Siliciano JD, Kajdas J, Finzi D et al. Long-term follow-up studies confirm the stability of the latent reservoir for HIV-1 in resting CD4+ T cells. Nat Med 2003; 9(6):727-728.

(3) Palmer S, Wiegand AP, Maldarelli F et al. New real-time reverse transcriptase-initiated PCR assay with single-copy sensitivity for human immunodeficiency virus type 1 RNA in plasma. J Clin Microbiol 2003; 41(10):4531-4536.

(4) Grinsztejn B, Bastos FI, Veloso VG et al. Assessing sexually transmitted infections in a cohort of women living with HIV/AIDS, in Rio de Janeiro, Brazil. Int J STD AIDS 2006; 17(7):473-478.

(5) Iwamoto M, Wenning LA, Petry AS, et al. Minimal Effect of Ritonavir (RTV) and Evavirenz (EFV) on the Pharmacokinetics (PK) of MK-0518. 46th Annual Interscience Conference on Antimicrobial Agents & Chemotherapy September 27-30, 2006. San Francisco, CA.

(6) Markowitz M, Nguyen BY, Gotuzzo F, et.al. Potent Antiretroviral Effect of MK-0518, a Novel HIV-1 Integrase Inhibitor, as Part of a Combination ART in Treatment Naive HIV-1 Infected Patients. 16th International AIDS Conference August 13-18, 2006. Toronto, Canada.

(7) Cooper DA GJRJ, Katlama Cetal. Results of BENCHMRK-1, a Phase III Study Evaluating the Efficacy and Safety of MK-0518, a Novel HIV-1 Integrase Inhibitor, in Patients with Triple-class Resistant Virus. 14^th^ Conference on Retroviruses and Opportunistic Infections February 25-28, 2007. Los Angeles, CA.

(8) Steigbigel R KPEJetal. Results of BENCHMRK-2, a Phase III Study Evaluating the Efficacy and Safety of MK-0518, a Novel HIV-1 Integrase Inhibitor, in Patients with Triple-class Resistant Virus. 14^th^ Conference on Retroviruses and Opportunistic Infections February 25-28, 2007. Los Angeles, CA.

(9) Grinsztejn B, Nguyen BY, Katlama C, et.al. Potent Antiretroviral Effect of MK-0518, a Novel HIV-1 Integrase Inhibitor, in Patients with Triple-class Resistant Virus. 46th Annual Interscience Conference on Antimicrobial Agents & Chemotherapy September 27-30, 2006. San Francisco, CA.

(10) Hughes MD. Analysis and design issues for studies using censored biomarker measurements with an example of viral load measurements in HIV clinical trials. Stat Med 2000; 19(23):3171-3191.

(11) Coffin J MFPSetal. Long-term Persistence of Low Level HIV-1 in Patients on Suppressive Antiretroviral Therapy. 13^th^ Conference on Retroviruses and Opportunistic Infections. February 5-8, 2006. Denver, CO.

# APPENDIX I: SPECIMEN COLLECTION, PROCESSING, AND SHIPMENT

1.0 OVERVIEW

All specimens will be collected per Section 6.0, Clinical and Laboratory Evaluations. Collection, processing, and shipment details for select specimens are below. For instructions regarding specimens not listed on this table, please refer to the Laboratory Processing Chart (LPC). All specimens generated from this protocol must be labeled, stored, and shipped according to the Laboratory and Data Management System (LDMS) guidelines unless otherwise specified.

2.0 TABLE

| Assay/Procedure | Tube Type or Specimen Type and Quantity | Derivative | Processing Instructions | Aliquots | Shipping Instructions |
| --- | --- | --- | --- | --- | --- |
| VIROLOGY |  |  |  |  |  |
| HIV-1 RNA UltraSensitive Roche Monitor Test  (real time) | 6 mL EDTA BLD | Plasma | Follow ACTG guidelines for processing. See section 3.0. | 2 x 1mL  BLD/EDT/PL2 | Ship 2 x 1 mL PL2 real time to JHU for HIV-1 RNA analysis. See section 4.0 for JHU shipping instructions. |
| HIV-1 RNA by SCA (real time and stored) | 50 mL EDTA BLD | Plasma | Whole blood samples should be collected in tubes containing K2EDTA (purple top). Plasma must be separated from whole blood and frozen at –80°C within 4 hours of sample collection. To collect plasma for analysis, the whole blood sample should be spun at 1350 x g for 15 minutes in a table top centrifuge. The plasma must then be removed very carefully without disturbing the buffy coat and/or mixing of white blood cells into the plasma. To prevent the removal of cells with plasma, leave about 0.5 mL of plasma behind on top of cells. This is very important because cellular debris will interfere with the single-copy assay analysis of the samples. If cells are inadvertently mixed with the plasma, re-spin the sample and remove the plasma without disturbing the cells  NOTE: when a sample is processed for the SCA, a sufficient layer of plasma must be left behind to ensure that absolutely no cells in the buffy coat or red blood cell layers are removed with the plasma. | 10 x 1.5 mL PL1 BLD/EDT/PL1 | Ship to:  Ann Wiegand/Sarah Palmer  1050 Boyles Street  Bldg. 535, Rm 109  Frederick, MD 21702 |
| Pre-ART Stored Plasma (if available) |  |  | See section 6.2.1 |  | Ship according to current BRI guidelines. See section 4.0. |
| Cell and Plasma Storage | 30 mL HEP BLD | Plasma  PBMC | Follow ACTG guidelines for processing. See section 3.0. | 5 x 1 mL PL2  BLD/HEP/PL2  As many cells @ 10x10^6^  BLD/HEP/CEL/DMS | Ship according to current BRI guidelines. See section 4.0. |
| IMMUNOLOGY |  |  |  |  |  |
| CD4+/CD8+ Cell Counts and Percentages | 4 mL EDTA BLD | N/A | N/A | N/A | Send whole blood to CLIA-certified laboratory with DAIDS IQA certification ambient. |
| Advanced Flow (activated CD4+ and CD8+cells) | 4 mL EDTA BLD | N/A | N/A | N/A | Send whole blood ambient by overnight carrier to the designated ISL. Samples must be processed within 30 hours after draw. Ship Monday through Thursday only. |
| PHARMACOLOGY |  |  |  |  |  |
| Trough PK | 4 mL PET EDTA BLD | Plasma | Place blood on ice immediately. Process within 30 minutes. Spin at 1500xg at 0-5°C for 10 min. Transfer plasma to a labeled 3.6 mL internally threaded Nunc cryotube (Nunc Part # 366524 or Fisher Part #12-565-172N). Store samples at -20°C until shipment to BRI. |  | Ship according to current BRI guidelines. See section 4.0. |

3.0 VIROLOGY CONSENSUS METHODOLOGY LINKS

- ACTG Laboratory Manual:

[http://aactg.s-3.com/LabManual.htm](http://aactg.s-3.com/LabManual.htm )

- ACTG Processing Guide Index:

<http://aactg.s-3.com/pub/download/labmanual/12-ALM-Specimen-Processing.pdf>

4.0 SHIPPING CONSENSUS METHODOLOGY LINKS

- Guidelines for Shipping Diagnostic Specimens:

<http://aactg.s-3.com/pub/download/labmanual/22-ALM-Diagnostic-Shipping-Guidelines.pdf>

- Guidelines for Shipping Infectious Substances:

<http://aactg.s-3.com/pub/download/labmanual/20-ALM-Infectious-Shipping-Guidelines.pdf>

- ACTG Specimen Repository (BRI) Shipping Guidelines:

<http://aactg.s-3.com/specrepos.htm>

- Outsourcing Laboratories (JHU/Quest) Shipping Instructions:

<http://aactg.s-3.com/laboutsourcing.htm>

- PSL Shipping Guidelines:

<http://aactg.s-3.com/members/inaphqac.htm>

APPENDIX II

DIVISION OF AIDS

AIDS CLINICAL TRIALS GROUP (ACTG)

SAMPLE INFORMED CONSENT

For protocol:

A5244, FINAL Version 1.0, 07/16/07: A Double-Blind, Randomized, Pilot Study to Measure the Effect of Treatment Intensification with a Potent Integrase Inhibitor, Raltegravir (MK-0518), on the Level of Persistent Plasma Viremia below 50 copies/mL in Subjects on Protease Inhibitor- or Non-Nucleoside Reverse Transcriptase Inhibitor-Containing Regimens.

SHORT TITLE: EFFECT OF TREATMENT INTENSIFICATION WITH RALTEGRAVIR (MK-0518) ON THE LEVEL OF PERSISTENT PLASMA VIREMIA BELOW 50 COPIES/ML, FINAL Version 1.0, 07/13/2007

INTRODUCTION

You are being asked to take part in this research study because you are infected with HIV, the virus that causes AIDS and you are currently on HIV medicines. This study is sponsored by the National Institutes of Health (NIH). The doctor in charge of this study at this site is: (insert name of Principal Investigator). Before you decide if you want to be a part of this study, we want you to know about the study.

This is a consent form. It gives you information about this study. The study staff will talk with you about this information. You are free to ask questions about this study at any time. If you agree to take part in this study, you will be asked to sign this consent form. You will get a copy to keep.

WHY IS THIS STUDY BEING DONE?

The use of anti-HIV therapy was originally considered a method to completely remove HIV and eventually “cure” those patients who were able to achieve undetectable viral loads.  However, for reasons that are still not fully understood, a low level of constant virus production (or a latent [concealed] reservoir) continues even with the use of therapy for HIV.  In this study, we are trying to find out if adding a new drug called raltegravir (MK-0518) to your HIV medicines will reduce the amount of HIV in your blood.

Most people who are told that they have “undetectable” viral loads still have HIV that can be measured in their blood using a sensitive test called the single copy assay (SCA). This is because regular viral load tests only measure down to 50 copies of HIV in each cc of blood whereas the SCA measures down to 1 copy in each cc of blood. In this study we will measure the amount of HIV in your blood using the SCA before and after you add raltegravir to your regular HIV medicines. We are also trying to find out if adding raltegravir to your HIV medicine causes any side effects or problems.

Raltegravir is an investigational drug and is not approved by the Food and Drug Administration (FDA). Raltegravir blocks HIV growth and lowers the amount of virus in the blood in the majority of infected people.

This study will enroll participants who are doing well on their HIV treatment and have undetectable viral loads that are less than 50 copies. Participants will be randomized (like the flip of a coin) to receive either raltegravir or placebo (like a sugar pill) for 12 weeks. After 12 weeks the participants will switch study drug (those on placebo will get actual raltegravir and those who were receiving raltegravir will be given placebo) for another 12 weeks. You will have an equal chance of being randomized to receive either raltegravir or placebo. Virus levels will be measured using the SCA. This will permit researchers to see what level of viral growth takes place in a person who is considered “well controlled” by our current standards and see if the addition of raltegravir changes anything. We will also be able to see if anything changes once the new drug is stopped. This may allow us to find out if adding raltegravir reduces the level of HIV in the blood below that which occurs with current therapy. This study will also look at whether it is safe to add raltegravir to other HIV medicines like the ones you are currently taking.

WHAT DO I HAVE TO DO IF I AM IN THIS STUDY?

If you agree to join this study, you will be asked to sign this consent form. After you have signed the form, you will be asked some questions and will undergo some tests at the screening visit to see if it is safe for you to join the study. The screening visit will take about 30-60 minutes.

At Screening

- You will be asked about your health and any medicine you have taken in the last 60 days.
- You will have a complete physical exam. The clinic staff will check your vital signs such as weight, height, temperature, blood pressure, breathing, and pulse.
- If you are a woman able to become pregnant, you will have a pregnancy test. Pregnant women cannot enter the study. If you become pregnant while on study, you will have to stop study treatment.
- You will have about 5 tablespoons of blood drawn for routine blood tests and for:
  - HIV viral load (the amount of HIV in your blood),
  - CD4+ and CD8+ cell count (the number of white blood cells that fight infection).
  - Viral load level using SCA.

If you do not enroll into the study

If you decide not to take part in this study or if you do not meet the eligibility requirements, we will still use some of your information. As part of this screening visit, some demographic (e.g., age, gender, race), clinical (e.g., disease condition, diagnosis), and laboratory (e.g., CD4+ cell count, viral load) information is being collected from you so that ACTG researchers may help determine whether there are patterns or common reasons why people do not join a study.

Pre-entry

If you have met all the requirements to enter the study, you will come to the clinic at least 24 hours after the screening visit for pre-entry evaluations. This visit will last about 30-60 minutes.

- You will be asked about your health and any medicine you have taken in the last 60 days.
- You will have a physical exam. The clinic staff will check your vital signs such as weight, temperature, blood pressure, breathing, and pulse.
- If you are a woman able to become pregnant, a urine or serum pregnancy test will be done if pregnancy is suspected.
- You will have about 7 tablespoons of blood drawn for routine blood tests and for:
- HIV viral load (the amount of HIV in your blood),
- CD4+ and CD8+ cells count (the number of white blood cells that fight infection),
- Viral load level using SCA (the single copy assay will be done in a batch at the end of the study),
- CD4+ and CD8+ activation tests (the percentage of your immune cells that have special markers that gives an idea of HIV disease activity),
- Extra blood for future immunology and virology tests.

Entry

At least 24 hours after pre-entry visit, you will come to the clinic for entry evaluations. This visit will last about 30-60 minutes. All of the evaluations you had at pre-entry will be repeated. In addition, some of the 7 tablespoons of blood drawn at this visit will be used for routine blood tests (blood chemistry and liver function tests). If you are a woman able to become pregnant, a urine or serum pregnancy test will be done whether or not pregnancy is suspected.

**You will be randomly assigned (as if by the toss of a coin) to the following groups:**

**Group A: you will add** raltegravir **to your other HIV medicines.**

**Group B: you will add a placebo (like a sugar pill) to your other HIV medicines.**

**The study drugs** raltegravir **and placebo are provided by the study and will be given to you at this visit. Your other HIV medicines will not be provided by the study. This is a blinded study, which means that you, your doctor, and the study staff will not know which study drug you are given.**

It is very important that you continue taking all your regular anti-HIV medicines as well as the study medicine. If you miss doses of the anti-HIV medicines, your virus may become resistant to the medicines, which means that the treatment may no longer work to control your HIV.

**On study evaluations**

**After you start taking the study medication, you will be asked to come to the clinic at weeks 2, 4, 10, 12, 14, 16, 22, and 24. At every visit your health will be checked and blood will collected to test your virus level using SCA. At entry, weeks 4, 12, and 16 visits you will be given your supply of study medicines to last until your next study visit.** Starting from the week 2 visit until the end of the study, you will bring your study medicines to the clinic to be counted and at weeks 4, 12, 16, and 24 visits the study staff will collect the remaining study medicines from you. On average, the on study visits will last about 30-60 minutes.

At weeks 4 and 16 you will have about 5 tablespoons of blood drawn for routine blood tests, liver function tests, and for:

- - HIV viral load (the amount of HIV in your blood),
  - Virus load level using SCA (the SCA will be done in a batch at the end of the study).

**At week 12, your study drug will be switched: If you were in Group A, you will switch and receive placebo. If you were in Group B, you will switch and receive** raltegravir**. You, your doctor, and the study staff will still not know what study drug you are receiving. At this visit, you will have the same evaluations as in the entry visit, but your medical and medication history will not be checked during these visits.**

At week 24, you will have all of the same evaluations as at the week 12 visit. In addition, if you are a woman able to become pregnant, a urine or serum pregnancy test will be done if pregnancy is suspected.

**You will be given the results of the pregnancy test (if applicable), CD4+ and CD8+ cell counts, viral load, and routine blood tests from these visits as soon as they become available.**

**At weeks 10, 12, 22, and 24, you will give an additional teaspoon of blood to check the study drug level in your blood.**

At weeks 4, 12, 16, and 24, you will be asked to fill in a questionnaire about how you take your anti-HIV drug. This questionnaire will take about 10-15 minutes to fill out.

If at any point during the study you are found to have a viral load value that is detectable (above 50 copies), you will be asked to come back in for another viral load test to make sure the first one was correct. If your viral load has not decreased enough or increased in the second viral load test, your doctor will take you off raltegravir.

If you stop taking the study medication before the end of the study, you will undergo all the same evaluations as in the entry visit, except extra blood will not be stored. You will also return for visits at week 12 and 24 (if you have not completed these visits already). The study medications will not be given to you at this visit; instead the clinic staff will collect the left-over study drug from you.

If you stop taking part in the study before the end of the study, you will undergo most of the same evaluations as in the entry visit. The study medications will not be given to you at this visit; instead the clinic staff will collect the left-over study drug from you.

If you are a woman able to become pregnant, a urine or serum pregnancy test will be done within 48 hours of study entry and at week 12 to make sure that you are not pregnant, and at any visit if pregnancy is suspected.

At the end of the study, you and your provider will receive a summary of the study’s results.

Other

If you agree, about 1 teaspoon of blood that is left over after all required study testing is done will be stored (with usual protectors of identity) and may be used for future ACTG-approved HIV-related research. Storage of leftover blood is not a requirement to participate in the study and you may withdraw your approval for the storage of your leftover blood, at anytime. These samples may be held for an indefinite length of time. We cannot ensure that you will be told of the results of the research done on these samples. Please indicate below whether you approve the use of your leftover blood.

*________ YES ________ NO*

HOW MANY PEOPLE WILL TAKE PART IN THIS STUDY?

About 50 people will take part in this study

HOW LONG WILL I BE IN THIS STUDY?

You will be in this study for about 24 weeks.

WHY WOULD THE DOCTOR TAKE ME OFF THIS STUDY EARLY?

The study doctor may need to take you off the study early without your permission if:

- the study is cancelled by the ACTG, the FDA, National Institutes of Health (NIH), the Office for Human Research Protections (OHRP), the drug company supporting this study, or the site’s Institutional Review Board (IRB). (An IRB is a committee that watches over the safety and rights of research subjects.)
- the Safety Monitoring Committee (SMC) recommends that the study be stopped early (the SMC is a group of experts appointed by the ACTG Scientific Committee who monitor the study)
- you are not able to attend the study visits as required by the study

The study doctor may also need to take you off the study drug(s) without your permission if:

- continuing the study drug(s) may be harmful to you
- you need a treatment that you may not take while on the study
- you are not able to take the study drug(s) as required by the study.
- you become pregnant.
- your viral load is confirmed to be ≥50 copies/mL on more than one measurement

If you must stop taking the study drug(s) before the study is over, the study doctor may ask you to continue to be part of the study and return for some study visits and procedures.

If I have to permanently stop taking study-provided drugs, or once I leave the study, how would the drugs be provided?

During the study:

If you must permanently stop taking study-provided drugs before your study participation is over, the study staff will discuss other options that may be of benefit to you.

After the study:

After you have completed your study participation, the study will not be able to continue to provide you with drugs you received on the study. If continuing to take these or similar drugs would be of benefit to you, the study staff will discuss how you may be able to obtain them.

WHAT ARE THE RISKS OF THE STUDY?

The drugs used in this study may have side effects, some of which are listed below. Please note that these lists do not include all the side effects seen with these drugs. These lists include the more serious or common side effects with a known or possible relationship. If you have questions concerning the additional study drug side effects, please ask the medical staff at your site.

There is a risk of serious and/or life-threatening side effects when non-study medications are taken with the study drugs. For your safety, you must tell the study doctor or nurse about all medications you are taking before you start the study and also before starting any new medications while on the study. Also, you must tell the study doctor or nurse before enrolling in any other clinical trials while on this study.

Risks of Drawing Blood

Taking blood may cause discomfort, bleeding, and bruising where the blood is drawn. Occasionally, there is swelling in the area where the needle enters the body and there is a small risk of infection. There is also a risk of lightheadedness, fainting, and blood clots.

Risks of Combination Antiretroviral Therapy

You are being asked to continue your combination antiretroviral medicines during the study. The use of combination antiretroviral medicines may be associated with certain side effects. You may ask your doctor for details on the side effects from your medications.

Immune reconstitution syndrome: In some people with advanced HIV infection, signs and symptoms of inflammation from other infections may occur soon after anti-HIV treatment is started.

The use of potent antiretroviral drug combinations may be associated with an abnormal placement of body fat and wasting. Some of the body changes include:

- Increase in fat around the waist and stomach area
- Increase in fat on the back of the neck
- Thinning of the face, legs, and arms
- Breast enlargement

Risks with Use of Raltegravir

The side effects listed below may be incomplete and our understanding of the safety of the drug may change during the course of this study. Several hundred people have received raltegravir in research studies.

The following side effects have been associated with the use of raltegravir in combination with other HIV drugs:

- Upset stomach (nausea, diarrhea, vomiting)
- Unusual tiredness
- Headache
- Dizziness
- Trouble sleeping, abnormal dreams
- Gas
- Itching
- Sleepiness
- Altered taste
- Loss/lack of appetite
- Constipation
- Abdominal pain
- Abnormal placement of body fat
- Muscle spasms
- Rash
- Stomach sticking out (abdominal distension)

Abnormal blood tests which have been seen in studies of raltegravir in combination with other HIV drugs include:

- Elevated liver enzymes, and bilirubin
- Increased levels of muscle enzymes
- Increased pancreas enzymes
- Low white blood cell counts or red blood cell counts
- Increases in cholesterol or triglycerides
- Worsening in a kidney function test

Some rare serious side effects have been seen in studies of raltegravir or placebo, in combination with other HIV drugs, and have each been reported in one or more participants.

- Pancreatitis (swelling of the pancreas)
- Excessive acid in the blood and body
- Kidney failure
- Stomach irritation
- Allergic reaction
- Heart attack
- Hepatitis (liver problems)

An increased rate of cancers was seen in people who took raltegravir with other HIV medications compared with people receiving placebo plus other HIV medications. These cancers were seen mostly in the first 3 months of treatment. Several were cancers that the people had before. The specific cancers were cancers seen previously in people with very sick immune systems. It is unknown if the increased rate of cancers was related to raltegravir use.

ARE THERE RISKS RELATED TO PREGNANCY?

Raltegravir may be unsafe for unborn babies. If you are having sex that could lead to pregnancy, you must agree not to become pregnant or make someone else pregnant. Because of the risk involved, you and your partner must use at least two methods of birth control, one of which must be a barrier method that you discuss with the study staff. You must continue to use both methods until 6 weeks after stopping your medicines. You must choose two of the birth control methods listed below:

- Condoms (male or female) with or without a spermicidal agent
- Diaphragm or cervical cap with spermicide
- IUD
- Hormone-based contraception

If you can become pregnant, you must have a pregnancy test before you enter this study. The test must show that you are not pregnant. If you think you may be pregnant at any time during the study, tell your study staff right away. If you become pregnant while on study, you must stop the study treatment.

Breastfeeding:

It is unknown whether the study drug passes through the breast-milk and may cause harm to your infant. If you breast-feed while on this study, you will have to stop the study treatment.

WHAT IF I BECOME PREGNANT DURING THIS STUDY?

If you become pregnant during the study, you must stop the study treatment but you will be asked to continue to come in for study visits. At these visits, you will have safety evaluations including routine safety tests, CD4 counts, viral loads and physical exams on the same schedule as described earlier in this consent. You will have about 2-3 teaspoons of blood drawn at each study visit for these tests.

You and your physician will decide what anti-HIV drug combination would be best for you to continue. This study will not provide care related to your pregnancy, the delivery of your baby, or the care of your baby. You must arrange for your care and your baby’s care outside of this study. This study will not provide your baby any anti-HIV drugs. Long-term follow-up is recommended for a baby whose mother takes anti-HIV drugs during pregnancy. The study staff will talk to you about your choices for long-term follow up.

ARE THERE BENEFITS TO TAKING PART IN THIS STUDY?

If you take part in this study, there may be a direct benefit to you, but no guarantee can be made. It is also possible that you may receive no benefit from being in this study. Information learned from this study may help others who have HIV.

WHAT OTHER CHOICES DO I HAVE BESIDES THIS STUDY?

Instead of being in this study you have the choice of:

- treatment with prescription drugs available to you
- treatment with experimental drugs, if you qualify
- no treatment

Please talk to your doctor about these and other choices available to you. Your doctor will explain the risks and benefits of these choices.

WHAT ABOUT CONFIDENTIALITY?

We will do everything we can to protect your privacy. In addition to the efforts of the study staff to help keep your personal information private, we have gotten a Certificate of Confidentiality from the U.S. Federal Government. This certificate means that researchers cannot be forced to tell people who are not connected with this study, such as the court system, about your participation. Also, any publication of this study will not use your name or identify you personally.

People who may review your records include the U.S. Food and Drug Administration (FDA), (insert name of site) IRB, National Institutes of Health (NIH), Office of Human Research Protection (OHRP), study staff, study monitors, drug company supporting this study, and their designees. Having a Certificate of Confidentiality does not prevent you from releasing information about yourself and your participation in the study.

Even with the Certificate of Confidentiality, if the study staff learns of possible child abuse and/or neglect or a risk of harm to yourself or others, we will be required to tell the proper authorities.

WHAT ARE THE COSTS TO ME?

Taking part in this study may lead to added costs to you and your insurance company. In some cases it is possible that your insurance company will not pay for these costs because you are taking part in a research study.

WHAT HAPPENS IF I AM INJURED?

If you are injured as a result of being in this study, you will be given immediate treatment for your injuries. The cost for this treatment will be charged to you or your insurance company. There is no program for compensation either through this institution or the National Institutes of Health. You will not be giving up any of your legal rights by signing this consent form.

WHAT ARE MY RIGHTS AS A RESEARCH SUBJECT?

Taking part in this study is completely voluntary. You may choose not to take part in this study or leave this study at any time. You will be treated the same no matter what you decide.

We will tell you about new information from this or other studies that may affect your health, welfare, or willingness to stay in this study. If you want the results of the study, let the study staff know.

WHAT DO I DO IF I HAVE QUESTIONS OR PROBLEMS?

For questions about this study or a research-related injury, contact:

- name of the investigator or other study staff
- telephone number of above

For questions about your rights as a research subject, contact:

- name or title of person on the Institutional Review Board (IRB) or other organization appropriate for the site
- telephone number of above

*SIGNATURE PAGE*

If you have read this consent form (or had it explained to you), all your questions have been answered and you agree to take part in this study, please sign your name below.

____________________________ _________________________________________

Participant’s Name (print) Participant’s Signature and Date

____________________________ _________________________________________

Participant’s Legal Guardian (print) Legal Guardian’s Signature and Date

(As appropriate)

____________________________ _________________________________________

Study Staff Conducting Study Staff’s Signature and Date

Consent Discussion (print)

____________________________ _________________________________________

Witness’s Name (print) Witness’s Signature and Date

(As appropriate)
